# Screening for Preterm Birth: Potential for a Metabolomics Biomarker Panel

**DOI:** 10.3390/metabo9050090

**Published:** 2019-05-07

**Authors:** Elizabeth C. Considine, Ali S. Khashan, Louise C. Kenny

**Affiliations:** 1 The Irish Centre for Fetal and Neonatal Translational Research (INFANT), Department of Obstetrics and Gynaecology, University College Cork, Cork T12 YE02, Ireland; a.khashan@ucc.ie; 2 School of Public Health, University College Cork, Cork T12 XF62, Ireland; 3 Department of Women’s and Children’s Health, Faculty of Health and Life Sciences, Institute of Translational Medicine, University of Liverpool, Liverpool L8 7SS, UK; louise.kenny@liverpool.ac.uk

**Keywords:** metabolomics, prediction, complex disease, heterogeneity, biomarkers, panel, preterm birth

## Abstract

The aim of this preliminary study was to investigate the potential of maternal serum to provide metabolomic biomarker candidates for the prediction of spontaneous preterm birth (SPTB) in asymptomatic pregnant women at 15 and/or 20 weeks’ gestation. Metabolomics LC-MS datasets from serum samples at 15- and 20-weeks’ gestation from a cohort of approximately 50 cases (GA < 37 weeks) and 55 controls (GA > 41weeks) were analysed for candidate biomarkers predictive of SPTB. Lists of the top ranked candidate biomarkers from both multivariate and univariate analyses were produced. At the 20 weeks’ GA time-point these lists had high concordance with each other (85%). A subset of 4 of these features produce a biomarker panel that predicts SPTB with a partial Area Under the Curve (pAUC) of 12.2, a sensitivity of 87.8%, a specificity of 57.7% and a *p*-value of 0.0013 upon 10-fold cross validation using PanelomiX software. This biomarker panel contained mostly features from groups already associated in the literature with preterm birth and consisted of 4 features from the biological groups of “Bile Acids”, “Prostaglandins”, “Vitamin D and derivatives” and “Fatty Acids and Conjugates”.

## 1. Introduction

### 1.1. Aim

Here we describe the first phase of a biomarker discovery project, the preclinical exploratory study, to identify a group of potential candidates for further investigation as early screening biomarkers of spontaneous preterm birth (SPTB) in asymptomatic pregnant women. In this proof of concept study the presence of missing values in the data is exploited by using a modification of a previously described [1], intuitively very simple method of imputation to create a situation where the case group of an extremely heterogeneous disorder approximates a homogenous disease group so that multivariate analysis techniques may be applied with some success.

### 1.2. Background of SPTB

Preterm birth, defined as birth occurring at less than 37 weeks’ gestation, is an archetype of a complex and heterogeneous disease or syndrome [2]. It is a multifactorial event with a variety of possible initiators and multiple etiologies. Heritability studies with twins show that genes account for approximately 30% of variation in preterm delivery [3]. SPTB occurs in approximately 10% of all pregnancies worldwide and accounts for approximately 70% of neonatal deaths and almost half of long-term neurological disabilities. Whether SPTB results from the premature activation of the normal labour pathway or labour by distinct means remains to be established. Despite decades of research the field is advancing slowly with as yet no reliable prediction method for asymptomatic women, nor any efficacious treatment method. 

Current prediction tests work only within a few days of delivery, and as such are more diagnostic than predictive, which gives very limited time for clinicians to decide on and institute the appropriate clinical course of action. Treatments for women at risk for preterm birth are based on the limited knowledge of the condition. Conventional preventative measures target patients who are deemed at possible risk of preterm birth owing to cervical insufficiency, bacterial infection, history of previous preterm birth, or those who are at imminent risk who are experiencing preterm labour (PTL) or preterm rupture of membranes (PPROM) or both. Interventions include administration of progesterone, cervical cerclage, bed rest and antibiotic use as first line preventative measures and tocolytic agents and again, progesterone as second line interventions. 

World Health Organisation (WHO) guidelines on screening [4] dictate that a treatment should exist before a screening test would be routinely used. It can be argued that this requirement should be circumvented in the case of preterm birth because routine screening and subsequent identification of high risk women provides the best opportunity for effective therapeutic interventions for preterm birth be developed. Successful identification of at-risk women at an early stage will be a major leap forward in the discovery science of preterm birth by allowing identification of the most informative population for pathophysiology understanding and intervention development.

The bio-fluid under study in this project is serum with the aim of ultimately developing a simple non-invasive routine screening blood to be performed as part of routine obstetrical visits to triage those at highest risk of subsequent preterm delivery. We identified only one previous study in the literature using LCMS to investigate the serum metabolomic profiles of pregnant women who subsequently gave birth preterm [5], although that study only had 3 cases of preterm delivery that were not complicated by other pregnancy disorders. We identified one study investigating the plasma metabolomic profiles of pregnant women who gave birth preterm, however in that study samples were collected at the time of threatened preterm labour, not at an asymptomatic stage [6]. To our knowledge this is the first study investigating the metabolomics serum profiles of asymptomatic, uncomplicated (for any other reason) pregnancies that ended in spontaneous preterm birth.

Given the heterogenous nature of SPTB its likely that a screening test will comprise a composite multi-marker panel, with different biomarkers accounting for SPTB’s various (as yet unknown) subgroups. From a clinical perspective, any putative screening test requires a high sensitivity, whereas a lower specificity, whilst not desirable may be acceptable. It would be clinically more important to identify as many at risk women as possible, even at the expense of a higher false positive rate rather than to miss some at risk women. The ‘ideal’ biomarker capable of predicting risk of SPTB early in pregnancy would be an endogenous metabolite with stable concentration in healthy pregnancy and with biological relevance to give clues to etiology to guide therapeutic intervention. It has been shown that the number of general disease biomarkers in serum, common to many very different diseases, may be larger than expected [7], so the identification of disease specific markers for unambiguous diagnosis of impending SPTB is desired.

### 1.3. Biomarker Discovery from Heterogeneous Disease

Successful biomarker discovery for heterogeneous disease such as SPTB requires more than double the number of cases and controls than for homogeneous disease and the probability of finding a true biomarker that exists in a heterogeneous dataset using 50 cases and 50 controls (our dataset size approximately) is only 15% [8]. Moreover, compounding the inherent clinical heterogeneity of SPTB, the case group in this study has a wide range of gestation lengths from 22 weeks to 36+ (<28 weeks: 4 cases; 28–≤31 weeks: 4 cases; 31+–<37 weeks: 41 cases). The extremely variable gestation at presentation, with the majority of cases presenting near term (20 of 41 cases in the 32–37-week category deliver after 36 weeks) means that only a small number of cases presented with the most severe phenotype. It is possible those cases delivering after 36 weeks, some 16 weeks following sampling at 20 weeks, may be much more like controls at a metabolomic level than like the other cases. This would seriously impact biomarker discovery and data analysis.

**Figure 1:**
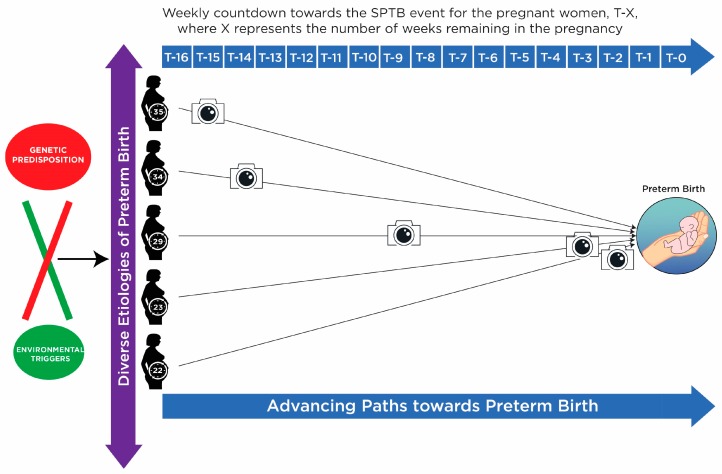
Schema depicting the molecular state of this spontaneous preterm birth (SPTB) dataset at metabolomics sampling time using a representative set of 5 patients. Diverse etiologies leading to SPTB ultimately converge in the final premature birth event. All women in the dataset were sampled at 20 weeks’ gestation, therefore at the same time relative to the start of their pregnancies. However, relative to the preterm birth event, the women were sampled at a variety of time points. For example, women who gave birth at 22 weeks were sampled at T-2 weeks and women who gave birth at 35 weeks were sampled at T-15 weeks. Snapshots (depicted by camera icons) of metabolomic activity taken at different stages preceding the SPTB even will be more divergent the further they are from the event.

As can be seen in the diagram (Figure 1), ultimately all pathways to SPTB eventually converge in the event of preterm birth. However, the sample timing leads to a variety of time-to-events ranging from 2 weeks to 16 weeks which results in the metabolomic snapshot being taken at various times in the disease process, or in some instances possibly before the disease process has commenced, at a metabolomic level at least. Intuitively this means that it is likely that the metabolomic snapshot has been taken at various points in the different etiological pathways resulting in a variety of differential features relative to controls. Therefore, there was little expectation of discovering a “signature” for SPTB that would be common to all or most cases, from this dataset. The expectation was that any biomarker candidates that were to be found in this dataset would represent some but not all cases, with a view to these candidates potentially contributing towards a combinatorial panel.

## 2. Methods

### 2.1. Patient Recruitment

The study cohort consisted of all women who participated in the Screening for Obstetric and Pregnancy Endpoints (SCOPE) study in Cork. Nulliparous healthy women with singleton pregnancies were recruited to the SCOPE study in Cork, Ireland. Enrolment into the study took place between November 2004 and January 2011, and the last SCOPE baby was delivered in Cork in August 2011. The aim of the SCOPE study was to develop early pregnancy screening tests to predict pre-eclampsia, SGA infants and spontaneous preterm birth. Details of the SCOPE study have previously been provided elsewhere [9]. In brief, pregnant women attending antenatal care settings such as maternity units, general practitioners, and outreach clinics and early pregnancy ultrasound appointments were invited to participate in the SCOPE study. Women who agreed to take part were interviewed and examined by a SCOPE research midwife at 15 ± 1 (visit 1) and 20 ± 1 (visit 2) weeks’ gestation. Detailed clinical and demographic data were collected at the first visit, including maternal characteristics such as age, body mass index (BMI), education level, ethnic origin, marital status, family income, previous pregnancy loss, participant birth weight and family history of obstetric complications and medical conditions. Women were excluded if they were at high risk of SGA, pre-eclampsia or spontaneous preterm birth because of underlying medical conditions. The data were entered into an internet-accessed central database with a complete audit trail (MedSciNet AB, Stockholm, Sweden, http://medscinet.com).

### 2.2. Study Design and Patient Demographics (Table 1)

This was a nested case-control study design with approximately equal numbers of cases and controls. Cases were individually matched to controls according to age within 5 years. Cases were also individually matched to a different set of controls to age within 5 years and BMI within 3 points.

### 2.3. Ethics

The SCOPE Ireland study was approved by the Clinical Research Ethics Committee of the Cork Teaching Hospitals [ECM5 (10)05/02/08) and is registered at the Australian New Zealand Clinical Trials Registry (ACTRN12607000551493) and all women provided written informed consent.

### 2.4. Sample Collection and Bio-Banking Procedures

Blood was processed within 3 h of collection to serum and stored in aliquots at −80 degrees Celsius for future analysis. No freeze thaw cycles were employed.

### 2.5. LCMS Analysis

LCMS analysis was carried out in both negative and positive ion modes at 2 time points: 15 weeks and 20 weeks’ as described by Dunn et al. [10] at Manchester University. For details please see Supplementary Materials.

### 2.6. Feature Annotation and Identification

All metabolite features were annotated according to level 2 of the MSI reporting standards applying PUTMEDID_LCMS, as previously described [11].

### 2.7. Data Preprocessing

Data preprocessing was carried out as described in the Supplementary Materials.

### 2.8. Data Analysis

Biomarker discovery was carried out on cases matched with controls on age only. Controls matched on age and BMI were not used for biomarker discovery for the following reasons:

(1) There was a risk of over matching due to BMI’s relationship with the outcome (SPTB) being controversial despite many investigations not established as a true confounder as opposed to a mediator.
(2) Matching can lead to bias that is offset by appropriate analysis, ie conditional logistic regression. However in this study the low stringency analysis used here is unlikely to be appropriate for matching.
(3) If the control group is too similar to the case group, the study may fail to detect the difference even if one exists. Since over 40% of the cases give birth very close to term from 36 weeks on, metabolomically speaking it is likely that they are very similar to controls. Matching to controls on age and BMI in this situation leads to overmatching as a risk.

However we do want to take advantage of this extra group of controls (matched on both age and BMI) which are composed of an entirely different set of individuals and so the performance of the candidates that are identified are assessed on the cases and the 2 sets of controls combined, so 49 cases and 104 controls to assess the performance of our candidate features.

#### 2.8.1. Incorporating Biological Knowledge—Explanation and Rationale

An unbiased approach (untargeted LCMS) was combined with a knowledge based approach [12] in the search for biomarker candidates. Candidate biomarker selection guided by expert knowledge is especially important in a disease such as SPTB where no efficacious treatment yet exists as potential therapies can be indicated by the biomarkers that give clues to disease etiologies [13].

The knowledge-based approach involved the following steps on the dataset generated in an unbiased way:

- Before data analysis begins obvious xenobiotics and exogenous metabolites (drugs, plant metabolites and others) and unidentified metabolites (Identified compounds were defined as compounds where the biological group was putatively identified at least even if the compound was not absolutely and uniquely identified) were removed from the unbiased and untargeted metabolomics dataset as these features would not form part of the biomarker panel.
- After data analysis domain knowledge was employed to guide the selection of plausible biomarker candidates from the top ranked features by:
  ○ Selecting those features that have supporting literature of their biological relevance and involvement in the disease process;
  ○ Selecting only those top ranked features with low or zero missing values for assessment in PanelomiX to avoid the caveats of the imputation method (identification of false positives);
  ○ Visually investigating the behaviour of each of the top ranked features across cases and controls according to the clinical requirements of a suitable biomarker candidate (stable in controls, perturbed in at least some cases) via scatter plots.

#### 2.8.2. Pretreatment—Explanation and Rationale

Let $x_{ij}$ be the measurements of levels for features (metabolites) i=1,2,…,m and samples (patients) j=1,2,…,n.

The samples fall into two groups, Group 1 is the case group and Group 2 is the control group. Let $C_{k}$ be the set of indices of the observations in group k for k=1,2.

Quality control (QC) correction consisted of calculating the relative standard deviation (RSD) of every feature using QC samples and removing from the dataset all those features with RSD>20%.

(1) $$RSD_{i}=\frac{s_{i}}{{\bar{x}}_{i}}$$

Here $s_{i}$ is the pooled standard deviation of metabolite i and ${\bar{x}}_{i}$ is the mean of metabolite i across both groups.

Filtering of all unidentified or exogenous features before pretreatment was carried out as screening biomarker candidates will consist of endogenous metabolites. Filtering was performed after QC correction and prior to any pretreatment so that only endogenous compounds remained that either had complete identification or identification by biological group.

Normalisation was carried out by dividing every row by the row mean. Where $x_{ij}^{N}$ represents the normalised values of the dataset, let

(2) $$x_{ij}^{N}=\frac{x_{ij}}{{\bar{x}}_{j}}$$

where ${\bar{x}}_{j}$ is the mean of each row, all metabolite measurements in a sample i.

Scaling was carried out by dividing every column by the mean of controls. This was performed on the normalised dataset.

(3) $$x_{ij}^{NS}=\frac{x_{ij}^{N}}{{\bar{x}}_{i2}}$$

where ${\bar{x}}_{i2}$ represents the mean of metabolite *i* in controls.

Imputation of cases and controls was performed separately. This was done based on their respective means for each feature in each group. Separate imputation is an uncommon method of imputation but not novel. We found only one other study that had performed separate imputation of cases and controls in metabolomics [1]. The imputation method performed here is a modification of the previous method. Where the previous method performed separate imputation of cases and controls using k-nearest neighbours, here the arithmetic mean of each feature, in each group, is used. Imputing case and control features separately according to their respective means serves to emphasise subtle differences in metabolite levels between the two groups, for example present only in a subset of the case group, rather than mask them. In the situation that that the missing value proportion for a particular feature is high, this approach has the potential to exaggerate the separation between the two groups and also exaggerate the within group similarity. For such instances, after data analysis, the missing data proportions of top ranked candidate features can be carried out so that these features, which have the possibility of being false positives, can be identified and scrutinised and removed from further analysis if necessary. In the situation that the missing value proportion for a particular feature is low, this imputation approach will preserve a subtle signal, such as coming only from a subset of the case group. This imputation method also prevents the contamination of a feature’s vector that has a very low variance in controls. If such a feature has a low proportion of missing values, their imputation based only on their group mean for that feature will preserve this low variance. Low variance in controls is a major requirement of our desired, ideal biomarker, that is, stable levels in healthy individuals.

For all values of i, for every missing value in column $x_{i}$ for Group 1 (cases)$\;mv_{i1}$, let

(4) $$mv_{i1}={\bar{x}}_{i1}$$

where ${\bar{x}}_{i1}$is the average measurement of metabolite i in Group 1 (cases).

And for all values of i, for every missing value in column $x_{i}$ for Group 2 (controls) $mv_{i2}$, let

(5) $$mv_{i2}={\bar{x}}_{i2}$$

where ${\bar{x}}_{i2}$ is the average measurement of metabolite i in Group 2 (controls).

Finally, the order in which the pretreatment steps are carried out is shown to influence the biomarker candidate lists produced in downstream analysis. It has been recommended that normalisation proceed imputation to reduce bias [14]. Many normalisation methods require a complete dataset with no missing values, the simple method that was used here however does not require a complete dataset. So the pretreatment steps were carried out in the following order: After QC adjustment normalisation was performed first, then scaling and finally imputation.

Steps were carried out in the following order:

QC Correction→Filtering→Normalisation→Scaling→Imputation

All steps were carried out using simple R commands and short R programs.

#### 2.8.3. Univariate Analysis (Fold Change)-Explanation and Rationale

The Fold Change (FC) for each feature (metabolite) i was calculated as the absolute value of the log2 of the ratio of averages of cases and controls and thus the features were ranked according to decreasing FC.

(6) $${FC}_{i}=\log_{2}\frac{{\bar{x}}_{i1}}{{\bar{x}}_{i2}}$$

The question of which univariate statistic to use should be based on the biological question of interest. The absolute changes in levels of a metabolite are what of interest for a clinically transferable combinatorial panel biomarker panel. *When absolute changes in levels of a feature are of interest, the fold change is superior to the t-test or its variations* [15]. Fold change is the simplest method for ranking differentially expressed features and was often the first method used in microarray data analysis. Fold change has been criticised as it does not control the variance as much as the t-test and related tests do, and as such is susceptible to outliers [16]. However, this criticism is not an issue for this study as features exhibiting outlier behaviour among the case samples are actively sought in this instance. In this heterogeneous dataset, a uniform shift among SPTB cases of a potentially informative feature was not imagined to be likely to exist. It is far more likely that for a particular feature associated with disease some cases will exhibit a shift (dysregulated level), therefore, features with large variances in cases were potentially of interest. Fold change ranking ranks those features with the largest variance in cases relative to the variance in controls. Fold change is also preferable when stringency is to be relaxed [17] and has been associated with high concordance and reproducibility [18]. Thus, for a starting point of feature selection for highly complex and heterogeneous disease dataset its advantages are intuitively obvious.

For comparison purposes non-parametric analysis Mann–Whitney U (MWU) test was also carried out on both datasets and features were ranked. No multiple testing criterion was applied.

#### 2.8.4. Multivariate Analysis—Explanation and Rationale

The data was then imputed as described in the pretreatment section above to deliberately amplify differences between cases and controls and at the same time amplify intra group similarities (to approximate homogeneity in the groups). Following imputation, multivariate analyses PLSDA + VIP were carried out using Metaboanalyst 4.0. Since the outcome variable (disease state) was used to impute the data, therefore deliberately incorporating bias, a predictive model was not derived. PLSDA was utilised however to orient the imputed data in multidimensional space to observe which features were most discriminating between the cases and controls. To assess the validity of the PLSDA model and the features selected according to it, permutation testing was performed (repeated 2000 times). Parameters employed for PLSDA and VIP analysis were the default settings of Metaboanalyst 4.0. Since data already had undergone pretreatment (normalisation, scaling and imputation) the normalisation and imputation steps were skipped in Metaboanalyst 4.0.

#### 2.8.5. Knowledge-Based Approach in Selecting Plausible Candidates

Supporting evidence of the top features found by both univariate and multivariate analysis involvement in preterm birth was sought in the literature.

#### 2.8.6. Missing Value Assessment for Avoiding Potential False Positives

Missing value proportions were assessed for both 15 week and 20 week datasets for cases and controls. Missing value proportions for each of the top ranked features according to each analysis were also calculated for both datasets. Only features with low missing values would be selected for biomarker panel assessment to try to minimise the risk of false positives.

#### 2.8.7. Visual Assessment of Features of Interest Distribution across Cases and Controls

Features of interest that had passed the previous two checks: supporting evidence in the literature and and low missing value proportion were then assessed visually, via scatter plots, for their distribution across case and control groups (in the original dataset before any imputation was applied). Box-and-whisker plots were deliberately not used for this purpose as the behaviour of the feature across individual samples was of interest and measures of central tendency were not of interest. This assessment was performed to check if the candidate features corresponded to or approximated the clinical requirements for a biomarker (stable in controls, perturbed in at least some cases) even before the deliberately biased imputation method had been applied.

#### 2.8.8. Panel Development

Panel experiments were performed using the PanelomiX toolbox (http://www.panelomix.net/) [19], which allows ROC analysis of panels by testing various combinations of markers based on the iterative combination of biomarkers and thresholds (ICBT) method. PanelomiX selects cut-offs for each biomarker to create the optimal panel performance. PanelomiX features a cross-validation procedure for panel verification and performs and shows the ROC curves of both the individual biomarkers and the panel using the pROC tool. The ROC curves of the cross-validation are built as the mean of centered predictions over the 10 CV folds. Panelomix has been previously used in metabolomics to find panels of discriminatory biomarker panels for lung cancer [20,21,22] and mild traumatic brain injury [23] and in proteomics to identify biomarker panels for bladder cancer [24] and mild traumatic brain injury [25].

#### 2.8.9. Alternative Biomarker Panels

For comparison purposes, the top ranked features from the 15 and 20 week datasets according to the Mann–Whitney U test were also tested in PanelomiX for their ability to build a biomarker panel that performed well upon cross validation.

## 3. Results

It is important to mention prior to describing the results of this unconventional data analysis that carrying out “conventional analysis” (*t*-test and non-parametric tests and also using conventional imputation methods followed by PLSDA + VIP analyses) on this dataset produced zero significant results. Specifically, this data was analysed in Metaboanalyst 4.0, and t-test and non-parametric tests identified zero significant features, even at and FDR cut off of 0.1. Using every available method of dealing with missing values in Metaboanalyst 4.0 (there are 9 in total: exclude features with missing values, replace by a small value, replace by mean/median/minimum, or estimate missing value using KNN/BPCA/PPCA/SVD impute) preceding PLSDA + VIP analysis led to models that were proven invalid by permutation testing, in every situation. Therefore, selecting features on the basis of any of these models would have been unreliable.

However, this result was expected from the outset, as explained in the introduction that this dataset was too heterogeneous and too small for global data analysis methods to work. At this point an investigator could erroneously believe that the dataset did not hold any valuable information pertaining to biomarker candidates for predicting SPTB.

### 3.1. Pretreatment: QC, Filtering, Normalisation, Scaling and Imputation

PCA score plot of samples and QC samples shows that QC samples cluster tightly together indiciating the reliability and stability of the data. (Figure S1 in Supplementary Materials).

QC treatment resulted in the removal of 192 features from the negative mode dataset and therefore the number of features (columns) of the dataset was reduced from 4055 to 3863. Filtering of exogenous and unidentified compounds further resulted in the number of features of the dataset being reduced from 3863 to 1348. Normalisation, scaling and imputation were carried out as described in the methods section in that order. (For visual representation of the flow of data through analysis, see Figures S2 and S3).

### 3.2. Results of Univariate and Multivariate Analysis on 15 Week Dataset Matched On Age Only

The top 20 metabolites ranked according to FC are presented in Table S1, of the Supplementary Materials for the 15-week dataset. The top 20 features ranked according to MWU test are presented in Table S2.

**Figure 2:**
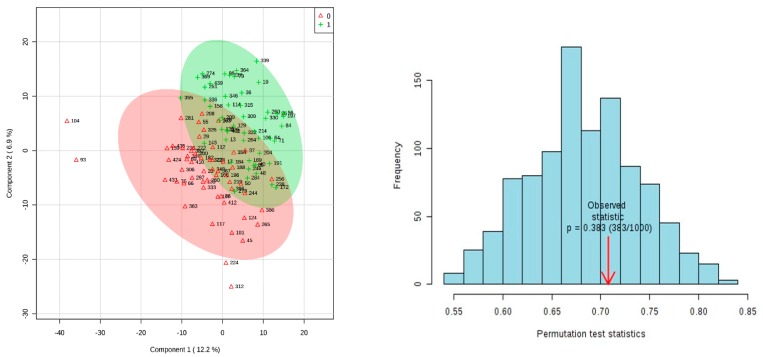
2-D PLSDA Score Plot from 15-week GA dataset with results of 2000 times permutation. Cases are green, controls are red. PLS-DA model validation by permutation tests based on prediction accuracy. The *p*-value based on permutation is *p* = 0.383 (383/1000).

PLSDA analysis was carried out on pretreated and imputed data using Metaboanalyst 4.0. A 2000 times permutation assessed the validity of the model. The results of the permutation test on the 15-week dataset (*p* = 0.383) showed that the model produced by PLSDA was invalid (Figure 2) so therefore selecting features on the basis of this model is unreliable. Biologically, this makes sense, as the pathway to SPTB may not have gotten under way yet at 15 weeks gestational age in many of the cases.

### 3.3. Results of Univariate and Multivariate Analysis on 20 Week Dataset Matched by Age Only

The top 20 metabolites ranked according to Fold Change are presented in Table 2 for the 20-week dataset. The top 20 features ranked according to MWU test are presented in Table S3.

PLSDA analysis was carried out on pretreated data using Metaboanalyst 4.0. A 2000 time’s permutation assessed the validity of the models produced from the 20 weeks dataset. VIP scores for the 20 week dataset are presented in Table 2.

**Figure 3:**
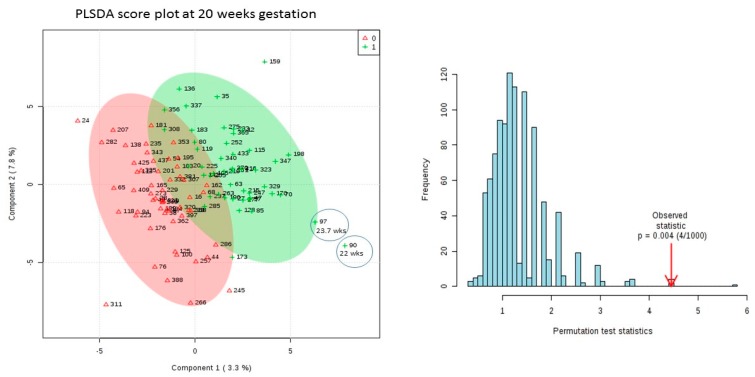
2-D PLSDA Score Plot from 20 week GA dataset (SPTB versus Age Matched Controls) with results of 2000 times permutation. Cases are green, controls are red. Earliest delivering cases (circled) are furthest from controls. PLS-DA model validation by 1000 permutation tests based on prediction accuracy. The *p* value based on permutation is *p* = 0.004 4/1000).

The results of the permutation test on the 20-week dataset (*p* = 0.004) showed that the model produced by PLSDA was valid (Figure 3) and no over-fitting was observed. Therefore, features ranked highest by VIP score according to this model are reliable. (Table 2).

Therefore, as expected the potential for predictive biomarker discovery was stronger at 20 weeks gestational age than at 15 weeks gestational age. For this reason, only those features from the 20 weeks dataset were investigated further for suitability as potential biomarker candidates.

### 3.4. Domain Knowledge to Guide Feature Selection for Panel Assessment

There is a high level (85%) of concordance between the lists generated by univariate (Fold Change) and multivariate (PLS-DA) analysis from the 20 weeks dataset. Out of the top 20 features extracted by univariate and multivariate analysis 17 are in common (Table 2). None of these top 17 features were unambiguously identified by PUTMEDID but they have been annotated so that their biological groupings have either been definitively or putatively identified as indicated in the third column of Table 3. Biological groups that were unambiguously identified and that were associated with preterm birth in the literature are presented below.

#### 3.4.1. Bile Acids and Preterm Birth

3 of the top ranked features at 20 weeks were in the biological group of Bile Acids (Figures S26–S28 in Supplementary Materials). The relationship between liver disease and adverse pregnancy outcomes is of great clinical importance. Total serum bile acids have previously been correlated with preterm birth [26]. Mawson suggests that preterm birth and related adverse birth outcomes occur due to liver dysfunction [27]. “With regard to the mechanism of preterm birth, it is proposed that cholestatic liver dysfunction and associated increases in circulating concentrations of retinyl esters and/or retinoic acids rupture the fetal membranes, inducing preterm birth and the characteristic features of the preterm infant, including retinopathy, necrotising enterocolitis and bronchopulmonary dysplasia.”

#### 3.4.2. Prostaglandins and Preterm Birth

Prostaglandins are well known for their involvement in preterm and term labour by inducing uterine contraction [28,29]. These results are consistent with this fact showing elevated prostaglandin levels in cases relative to controls (Figure S38 in Supplementary Materials).

#### 3.4.3. Vitamin D and Preterm Birth

3 of the top ranked features at 20 weeks are in the biological group of Vitamin D (and derivatives) (Figures S29, S35, S37 in Supplementary Materials) and 1 is putatively identified as Vitamin D and derivatives (Figure S24 in Supplementary Materials). Three of these features show a pattern of elevation in controls relative to cases (Figures S24, S29, and S35 in Supplementary Materials). This is consistent with many findings in the literature that Vitamin D insufficiency is associated with risk of preterm birth. Both individual studies [30,31] and meta-analyses [32,33] find an association between Vitamin D insufficiency and preterm birth.

#### 3.4.4. Fatty Acids and Preterm Birth

4 of the top ranked features at 20 weeks were either putatively (Figures S24, S31, S39 in Supplementary Materials) or definitively (Figure S33 in Supplementary Materials) identified as Fatty Acids. Perturbed fatty acid levels have previously been reported as being associated with an increased risk of preterm birth and preterm labour [5,6,34,35,36].

### 3.5. Missing Value Assessment to Guide Feature Selection for Panel Assessment

The level of missing values in each feature was used to guide candidate selection for panel assessment to offset the limitation of this biomarker discovery method being biased towards identifying features with high levels of missing values.

Missing Values: 15-week dataset (Table S4).

The top 20 ranked features by FC, from the 15 week dataset, contained approximately 24% missing values, with similar proportions of missing-ness in cases and controls for each feature, Table S1. The top ranked features by MWU test, from the 15 week dataset contained 10% missing values with similar proportions of missing-ness in cases and controls, Table S2.

Missing values: 20-week dataset (Table S5).

The levels of missing-ness of top 17 features from the 20-week dataset are presented Table 2. Overall this group of 17 features had 18% missing values with approximately equal proportions in cases and controls for each feature. Individually, some of these top ranked features, as would be expected due to the imputation method employed, have quite high levels of missing-ness with approximately similar levels of missing-ness in cases and controls. The top ranked features by MWU test, from the 20-week dataset contained 11% missing values with similar proportions of missing-ness in cases and controls, Table S3.

### 3.6. Visual Examination to Guide Feature Selection for Panel Assesment

The distribution of these 17 features across cases and controls was presented by scatter plots. (Supplementary Materials, Figures S24–S40). Guided by selecting features whose (1) biological grouping was unambiguously identified and associated with preterm birth in the literature, had (2) low missing value proportions, and (3) exhibited distributions across the samples that are most consistent with an ideal biomarker candidate, led to the selection of 4 features for assessment in a candidate biomarker panel. These were a bile acid, a prostaglandin, a fatty acid and a vitamin D and derivatives (Figures S27, S33, S37, and S38 in the Supplementary Materials).

**Figure 4:**
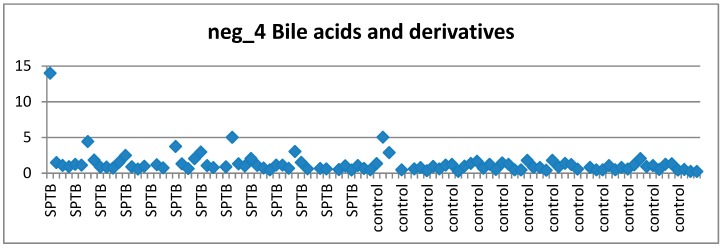
Distribution of feature neg_4 (Bile Acid) across cases and controls.

**Figure 5:**
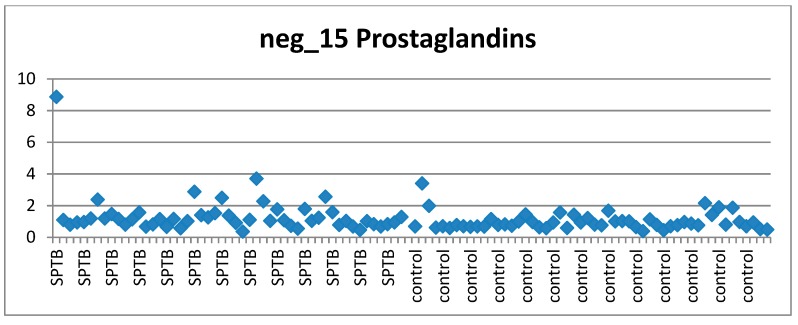
Distribution of feature neg_15 (Prostaglandin) across cases and controls.

As an example, the scatter plots of two of these features, satisfying all 3 criteria, are shown here. The bile acid (Figure 4) and the prostaglandin (Figure 5) exhibited (mostly) stable concentration in controls and perturbed in (at least some) cases.

### 3.7. Biomarker Panel Assessment with PanelomiX

#### 3.7.1. Panel A: 4 Features from 20 Week Dataset Ranked Highly by Fold Change/PLSDA and VIP Preceded by Separate Imputation

Judicious feature selection according to the previous criteria amounted to 4 features (Panel A) indicated with an asterisk in the penultimate column of Table 2. This panel contained a Bile Acid, a Prostaglandin, a Vitamin D and derivatives and a member of Fatty Acids and Conjugates. This panel’s performance was tested on the 20 week dataset that consisted of cases matched with both sets of controls (49 cases and 104 controls). PanelomiX revealed that the panel of all 4 features had the highest performance, (compared with any combination of fewer than 4 of the features) with a pAUC of 12.2, a sensitivity of 87.8%, a specificity of 57.7% and a *p*-value of 0.0013 upon 10-fold cross validation (Table 3).

#### 3.7.2. Panel B: 6 Features from 20 Week Dataset Ranked by Mann–Whitney U test

For comparison purposes the top ranked 6 features according to the non-parametric Mann–Whitney U test (also with low missing values) from the 20 week dataset were assessed in PanelomiX for their ability to create a biomarker panel. This panel’s performance was tested on the 20 week dataset that consisted of cases matched with both sets of controls (49 cases and 104 controls). The best panel produced by these features performed reasonably well with a pAUC of 10.3 a sensitivity of 77.6 and a specificity 57.7 and a *p*-value 0.09158 upon cross validation (Table 3).

#### 3.7.3. Panel C: 4 Features from 15-Week Dataset Ranked in Top 20 by Fold Change

A panel that performed well from the 15 week dataset was not expected. Nonetheless for the purposes of comparison, the features ranked highest according to fold change from the 15 week dataset were analysed for their ability to form a biomarker panel. This resulted in a panel that did not withstand cross validation (*p*-value = 0.24057) (Table 3).

#### 3.7.4. Panel D: 4 Features from 15-Week Dataset Ranked by Mann–Whitney U test

The top ranked features by MWU test from the 15 week datastet were also assessed for their ability to form a biomarker panel. Again this analysis resulted in a panel that did not withstand cross validation (*p*-value = 0.59576) (Table 3).

#### 3.7.5. PanelomiX Results Summary

The best performing panel (Panel A) consisted of features from the Biological Groups of Bile Acids, Vitamin D and derivatives, Prostaglandins and Fatty Acids and Conjugates (Table 4). The best performing candidate biomarker of this panel according to PanelomiX was the Bile Acid.

Logistic regression was also performed for all 4 panels by PanelomiX software. The results of logistic regression can be found in Table S6 in the Supplementary Materials.

## 4. Discussion

In this study we attempted to mitigate the extreme heterogeneity in our disease group and succeeded in extracting a group of plausible clinical biomarker candidates for assessment at the targeted stage. These candidates have strong supporting evidence in the literature of their involvement in preterm labour, term labour or parturition, and they perform well upon cross validation (at least for this dataset) according to biomarker panel assessment with PanelomiX.

We are reluctant to rely heavily upon the performance that was observed on cross validation in PanelomiX, as we did not yet have the opportunity to validate our candidates in an external dataset, but we propose that the performance in PanelomiX, along with evidence in the literature are promising indicators of the potential of such a panel.

A limitation of our study is that due to the heterogeneity of this disease all possible subtypes may not be represented by this dataset so when these candidates are generalised to the whole population they may experience a reduction in sensitivity. Also choosing the top 20 is an arbitrary cut off and it is possible that there are other useful features beyond this cut-off.

It remains likely that larger cohorts of patients and other novel data analysis methods that respect the heterogeneity of the PTB syndrome may turn up more biomarker candidates to add to the list of candidates informing the final biomarker candidate panel.

We employed the shortest analysis pipeline and most simple analysis methods possible to reduce the likelihood of the accidental introduction of error and/or bias and to preserve subtle signals that may represent only subgroups of cases.

The high level of concordance between Fold Change ranked features and PLSDA VIP score ranked features at 20 weeks is likely to be due to the fact that Fold Change is susceptible to outliers and the imputation method employed biases in favour of outliers. So it is possible that both methods accept and highlight the situation where a feature might exhibit perturbation in only a subset of case samples which is precisely the features we were interested in uncovering. It is worth restating that this is not a novel imputation method [1] but that in this study the original method is adapted slightly and exploited it for its utility in approximating homogeneity in each group.

In the original use of this method of imputation [1], how it affects the data was not discussed. We discussed in the methods section how this imputation, for a feature with a high level of missing-ness, has the potential to artificially increase within group similarity and decrease between group similarity. This can give rise to false positives. To counteract this caveat, features with a high level of missing-ness that are ranked highly by the method, can be investigated post hoc and removed from further analysis. However for a feature with a low level of missingness, this imputation method will conserve low variance if it exists and conserve subtle signals only coming for a portion (subset) of a group if it exists. This means that for the feature of interest to us with stable levels of the metabolite in healthy individuals (low variance in controls) and a subset of dysregulated cases in the disease group, the signal will be preserved.

Among the 17 features identified by univariate and multivariate analysis at 20 weeks, 5 features have high levels of missingness (>30% missing values in cases or controls or both). Those with the highest levels of missingness were features from the following biological groups: octadecanoids; bile acids and derivatives; phosphoinositols; vitamin D3 and derivatives; and amino ketones/amino acids/acyl glycines/hydroxy fatty acids.

Small signaling molecules hold potential as powerful biomarkers as they may identify subtle changes in the metabolome that occur prior to the detection of a gross phenotypic change reflecting disease [37]. Bile acids [38], prostaglandins [39], phosphoinositols [40], and vitamin D and derivatives [41] have all been reported in the literature as being involved in signaling and are known difficult to detect due to their low concentrations in biological samples. These compounds would be likely to suffer from missing-ness and consequently be susceptible to their signals being masked by imputation, especially in the situation where they are only dysregulated in a subgroup of cases.

According to the results, the decision not to analyse the data matched on age and BMI was appropriate. BMI does indeed a correlate with the exposures of interest revealed in the biomarker discovery phase of our study: serum vitamin D levels [42,43], prostaglandin levels [44] and bile acids [45]. Also, whether BMI is a predictor of SPTB is inconclusive [46,47,48,49].

We observed that SPTB occurring within 4 weeks of the sample testing time may be easier to detect using metabolomics profiling. This is indicated on the PLSDA plot from the 20-week sampling (Figure 3) showing that samples from the 2 patients that were earliest to deliver (gestational ages at delivery of 22 weeks and 23.7 weeks) are furthest from controls.

Following on from this preliminary study, we propose that at Phase 2, the same cases and controls are used for the for absolute quantification of the selected group of candidate biomarkers from Phase 1 via targeted approaches such as multiple reaction monitoring mass spectrometry (MRM-MS) [15]. After confirming the differential abundance of the candidate biomarkers at the qualification phase (Phase 2), the verification stage is next where a multi-marker panel should be built, and its performance tested internally using cross validation and also externally using an independent validation dataset (Phase 3).

We have two main conclusions from this study, one regarding SPTB and one regarding biomarker discovery in heterogeneous disease in general.

From our findings we assert that a combinatorial biomarker panel for the prediction of SPTB is both possible and feasible and that such a panel could potentially be readily accepted by the medical community without suffering problems of “abstraction” due to black box model building methods or from being composed of features that are not readily idenfitiable with meaningful biological mechansim. Such a panel would likely comprise bile acids, prostaglandins, fatty acids and vitamin D and derivatives at a minimum.

We also suggest that for highly heterogeneous datasets, typical data analysis methods used in metabolomics biomarker discovery studies may be more suited to finding global differences and analysis of homogenous datasets. Therefore, de facto analysis methods may fail to identify candidates that individually only represent a portion of the dataset (or the disease population) but together may produce a useful combinatorial panel.

In the case of heterogeneous disease, extracting signals representative of subgroups of the population and subsequently combining them into combinatorial panels may produce a signature that is more likely to be generalisable and therefore perform well in independent validation cohorts.

The potential criticisms of this study are:

(1) This imputation method is biased towards identifying features with high missing values,
(2) This imputation method is biased towards artificial separation of cases and controls by increasing inter group similarity and decreasing intra group similarity so that good performance of PLSDA upon permutation is expected,
(3) Lack of an independent validation cohort, and
(4) This imputation method could lead to the identification of potential of false positives.

We attempt to offset these limitations by

(1) Selecting at the end of our analysis those features with low or zero missing values for assessment in biomarker panels using the PanelomiX program;
(2) Showing that for the 15-week dataset despite the exact same imputation and analysis method being used as for the 20 week dataset this does not lead to a PLSDA model showing separation of cases and controls that stands up to permutation.
(3) Although at this point we do not validate in an independent cohort the features are assessed in PanelomiX by 10-fold cross validation and perform well with a *p*-values of 0.0013. Also the assessment occurred on the dataset with both sets of controls so an extra 49 healthy subjects who weren’t involved in the discovery of the candidate features.
(4) The fact that MWU test top ranked features at 20 weeks are tested by PanelomiX and create a panel that performs not as well upon cross validation as those found through fold change and imputation method is an argument against the method being more susceptible to false positives.

## Figures and Tables

**Table 1:** Demographic information.

	Controls Age Matched within 5 Years (15 Weeks)	SPTB (15 Weeks)	Controls Age (within 5 Years) and BMI Matched within (3 Points) (20 Weeks)	Controls Age Matched (within 5 Years) (20 Weeks)	SPTB (20 Weeks)
Number of samples	56	50	49	55	49
Age of patients (average)	30.33	30.18	30.21	30.35	30.16
BMI of patients (average)	24.54	25.13	24.53	24.75	24.78
Gestational Age at Delivery (Weeks)	>41	22–28 (4 cases) 28–31 (4 cases) 32 ≤ 37 (42 cases)	>41	>41	22–28 (3 cases) 28–31 (4 cases) 32 ≤ 37 (42 cases)
Pathway to Delivery	Term	PTL (23) PPROM (27)	Term	Term	PTL (23) PPROM (26)

BMI: Body Mass Index; PTL: preterm labour; PPROM: preterm premature rupture of membranes.

**Table 2:** Ranked results of univariate and multivariate analysis on 20 weeks GA dataset.

Rank	Feature ID	Biological Grouping	Ranked by Fold Change	Direction of Dysregulation in Cases	VIP Score	VIP Ranking	% Missing Values in Cases	%Missing Values in Controls	Features in Panel A
1	neg_1	VITD/GLU/FA	1.761188	DOWN	4.2515	6th	4	4	
2	neg_2	DIAG	1.696499	DOWN	4.0385	7th	55	42	
3	neg_3	BA	1.672851	UP	6.6187	1st	33	40	
4	neg_4	BA	1.669295	UP	6.5837	2nd	10	5	*
5	neg_5	BA	1.64909	UP	6.3849	3rd	33	42	
6	neg_6	VITD3	1.62416	DOWN	3.7802	11th	35	29	
7	neg_7	AK/ALD	1.560826	DOWN	3.5345	14th	0	0	
8	neg_8	GLP/HFA	1.541056	DOWN	3.4536	15th	55	56	
9	neg_9	AG/AK/AA/IND	1.539377	DOWN	3.4467	16th	0	0	
10	neg_10	FA	1.500618	UP	4.9245	4th	8	7	*
11	neg_11	AG/AK/AA	1.485738	DOWN	3.216	19th	0	0	
12	neg_12	VITD	1.481957	DOWN	3.1991	20th	0	0	
13	neg_13	STG	1.458342	UP	4.5086	5th	4	5	
14	neg_14	VITD	1.406144	UP	3.9951	8th	20	9	*
15	neg_15	PG	1.400314	UP	3.9378	9th	0	2	*
16	neg_16	AK/AG/AA/HFA	1.392332	UP	3.8593	10th	29	22	
17	neg_17	OCT	1.383006	UP	3.7675	12th	31	40	

Abbreviations: AA: amino acids; AG: acyl glycines; AK: amino ketones; ALD: aldehydes; BA: bile acid; DIAG: diacylglycerophosphoinositols; FA: fatty acid; GLU: gluconorides; GLP: glycerolipids; HA: hydroxy acids; HFA: hydroxy fatty acids; IND: indoles; OCT: octadecanoids; PG: prostaglandins; STG: stigmasterols; VITD: vitamin D and derivatives. *denotes features in Panel A.

**Table 3:** PanelomiX Results Summary.

Panel	Dataset (GA in Weeks)	Feature Selection Method	% Missing Values	# Features in Final Panel	pAUC	% Specificity (95%CI)	% Sensitivity (95%CI)	*p*-Value upon 10 Fold cv
Panel A	20 vs All Controls	FC and PLSDA +VIP	7	4	12.2 (8.4–15.9)	57.7 (49.0–67.3)	87.8 (77.6–95.9)	0.0013
Panel B	20 vs All controls	MWU	1	5	10.3 (7.4–14.0)	57.7 (48.1–67.3)	77.6 (65.3–89.8)	0.09158
Panel C	15	FC and PLSDA+VIP	4	4	10.4 (6.1–15.2)	73.2 (60.7–83.9)	75.5 (63.3–87.8)	0.24057
Panel D	15	MWU	3	4	9.7 (5.8–14.9)	62.5 (50.0–75.0)	77.6 (65.3–87.8)	0.59576

FC: Fold Change; MWU: Mann–Whitney U test; PLSDA and VIP: partial least squares discriminant analysis and variable importance projection.

**Table 4:** Table of features of the top performing panel found by FC and PLSDA/VIP ranking preceded by separate imputation for detection of SPTB at 20-weeks’ gestation.

Panel A Features	Biological Group	Fold Change	VIP Score	MWU- *p* Value	% Missing Values	Top Performing Feature in PanelomiX
neg_4	BA	1.669295	6.5837	0.0171	6	****
neg_10	FA	1.500618	4.9245	0.0064	7	-
neg_14	VITD	1.406144	3.9951	0.2599	12	-
neg_15	PG	1.400314	3.9378	0.0062	3	-

BA: bile acid; FA: fatty acid; VITD: vitamin D; PG: prostaglandin. ****denotes the top performing feature of the panel according to Panelomix.

## Supplementary Materials

The following are available online at https://www.mdpi.com/2218-1989/9/5/90/s1, Figures S1–S40, and Tables S1–S5.
