# Supplementary material for: Screening for Preterm Birth: Potential for a Metabolomics Biomarker Panel
Source: Metabolites. 2019 May 7;9(5):90. doi: 10.3390/metabo9050090 (PMC6572582; doi:10.3390/metabo9050090)
Supplement: Supplementary file 1 [file metabolites-09-00090-s001.pdf]

SUPPLEMENTARY MATERIALS

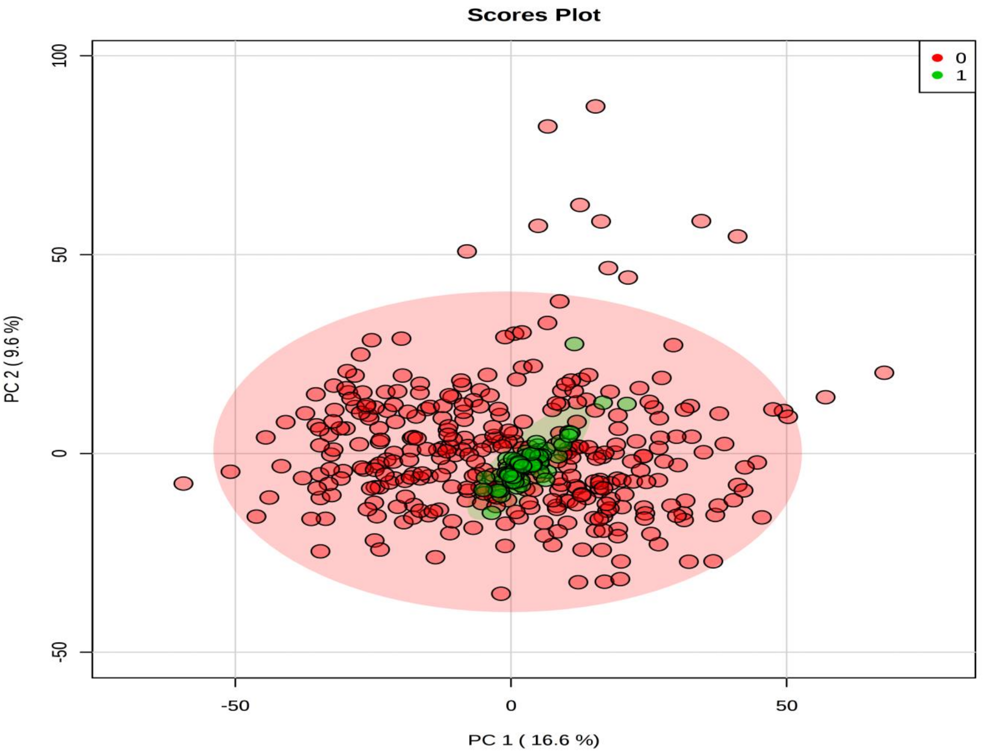

Figure S1: PCA score plot of patients (red) and QC samples (green). QC samples are seen to cluster tightly together indicating that the data is of good quality.

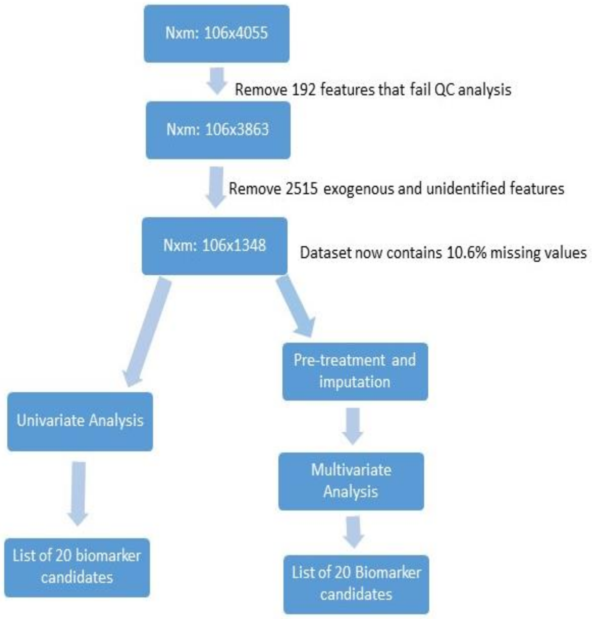

Figure S2. Flow of data from raw data stage through data analysis to biomarker candidate lists: 15 weeks dataset: 106 samples (50 cases and 56 controls) x 4055 features.

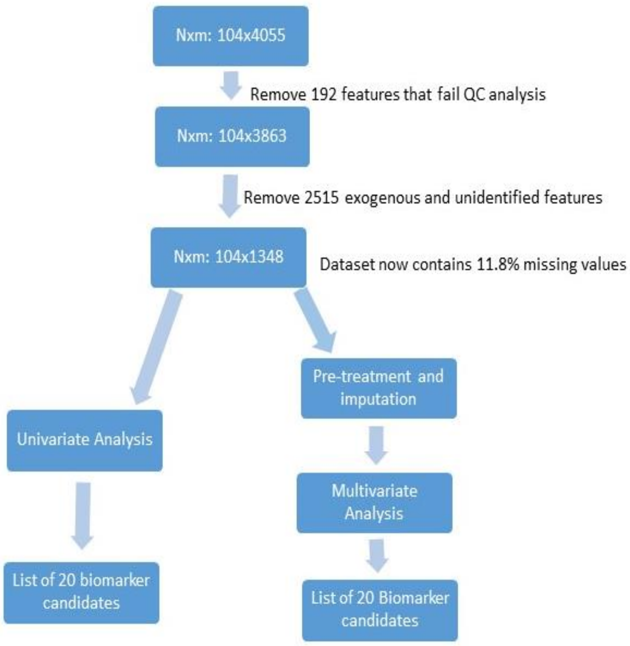

Figure S3. Flow of data from raw data stage through data analysis to biomarker candidate lists: 20 weeks dataset: 104 samples (49 cases and 55 controls) x 4055 features.

Table S1: Ranked results of univariate (Fold Change) analysis on **15** weeks GA dataset. (VIP scores are not reported as the results of the permutation showed that the PLSDA model was invalid.)

Rank	Feature	Fold Change	Log 2 Fold Change	Direction of Dysregulation in Cases	% missing values cases	% missing values controls	Biological Grouping	Panel C
**1**	neg_18	2.440485	1.287168	UP	39	45	BA	
**2**	neg_19	2.25624	1.173921	UP	37	46	BA	
**3**	neg_20	0.514199	0.9596	DOWN	2	5	PL	*
**4**	neg_21	0.563609	0.827234	DOWN	41	50	FA	
**5**	neg_22	0.594372	0.750562	DOWN	6	5	AA	*
**6**	neg_23	0.603438	0.728722	DOWN	18	20	PL	
**7**	neg_24	0.609538	0.714212	DOWN	41	32	SP/PP/AA/DA	
**8**	neg_25	0.609667	0.713906	DOWN	41	39	DIAG	
**9**	neg_26	1.508704	0.59331	UP	43	30	C21-STR	
**10**	neg_27	0.67683	0.563134	DOWN	35	25	ARA/HA/ALC/PO/CAT	
**11**	neg_28	0.693309	0.52843	DOWN	0	0	PL	*

**12**	neg_29	1.440332	0.526402	UP	24	18	BA	
**13**	neg_30	0.695696	0.523471	DOWN	29	21	PL	
**14**	neg_31	0.698078	0.51854	DOWN	39	43	VITD &d	
**15**	neg_32	0.701671	0.511133	DOWN	0	0	PL	
**16**	neg_33	0.702933	0.50854	DOWN	0	2	PL	
**17**	neg_34	1.417347	0.503194	UP	45	23	FA & c/STR &d	
**18**	neg_35	0.706089	0.502078	DOWN	0	0	PL	
**19**	neg_36	1.41598	0.501801	UP	39	52	PTR	
**20**	neg_37	0.708661	0.496832	DOWN	4	7	MOAG	*

Table S2: Non-parametric test MWU ranked top 20 features from 15 week dataset

Feature	P-VALUE	FDR	% mv in cases	%mv in controls	ID/Biological Grouping	Panel D
neg_38	0.000951	0.83074	0	0	HFA	*
neg_39	0.002162	0.83074	0	0	IND	*
neg_40	0.002619	0.83074	39	59	PTR	
neg_41	0.004049	0.83074	33	33	GLP	
neg_42	0.004952	0.83074	16	12	VIT D	
neg_43	0.007468	0.83074	4	8	MOAG	*
neg_44	0.010098	0.83074	2	6	PL	*
neg_45	0.010479	0.83074	24	16	VITD/BA/PP	
neg_46	0.010674	0.83074	37	59	PTR	
neg_47	0.010674	0.83074	0	0	DIAG	
neg_48	0.0117	0.83074	2	0	MOAG/PL	
neg_49	0.0117	0.83074	6	6	DIAG	
neg_50	0.012356	0.83074	10	24	N_GLSP	
neg_51	0.013282	0.83074	0	0	VITD	
neg_52	0.014016	0.83074	12	6	PL	
neg_53	0.014269	0.83074	0	0	DIAG	
neg_54	0.014786	0.83074	0	0	DIAG	
neg_55	0.014786	0.83074	0	0	DIAG	
neg_56	0.014786	0.83074	2	0	DIAG	
neg_57	0.015871	0.83074	0	0	PL	

Table S3: non-parametric test MWU ranked top 20 features from 20 week dataset

FEATURE	p-value	FDR	% MV in cases	% mv in controls	ID/Biological Grouping	Panel B
neg_58	0.000726	0.71503	0	0	HFA	*

neg_59	0.003315	0.71503	2	2	VITD	*
neg_60	0.003757	0.71503	0	0	FALD	*
neg_61	0.003836	0.71503	20	15	PL	
neg_62	0.005877	0.71503	28	29	PL	
neg_63	0.005995	0.71503	26	29	N-ACLA	
neg_64	0.006237	0.71503	18	25	PP/IND & d	
neg_65	0.006237	0.71503	0	2	PG	*
neg_66	0.006882	0.71503	0	0	HFA/FALD	*
neg_67	0.007018	0.71503	0	0	PL	*
neg_68	0.007587	0.71503	0	5	FA & c	
neg_69	0.008517	0.71503	2	0	FA & c	
neg_70	0.008849	0.71503	30	40	OCT	
neg_71	0.008849	0.71503	44	22	SPM	
neg_72	0.009192	0.71503	18	11	FA & c	
neg_73	0.009369	0.71503	0	0	FA & c	
neg_74	0.009548	0.71503	0	0	PA/A/PO	
neg_75	0.009548	0.71503	22	29	GLP	
neg_76	0.010104	0.71685	12	16	DIAG	

15 weeks gestational age dataset: Top features according to Fold Change Ranking

Figure S4: neg_18 at 15 weeks in cases and controls

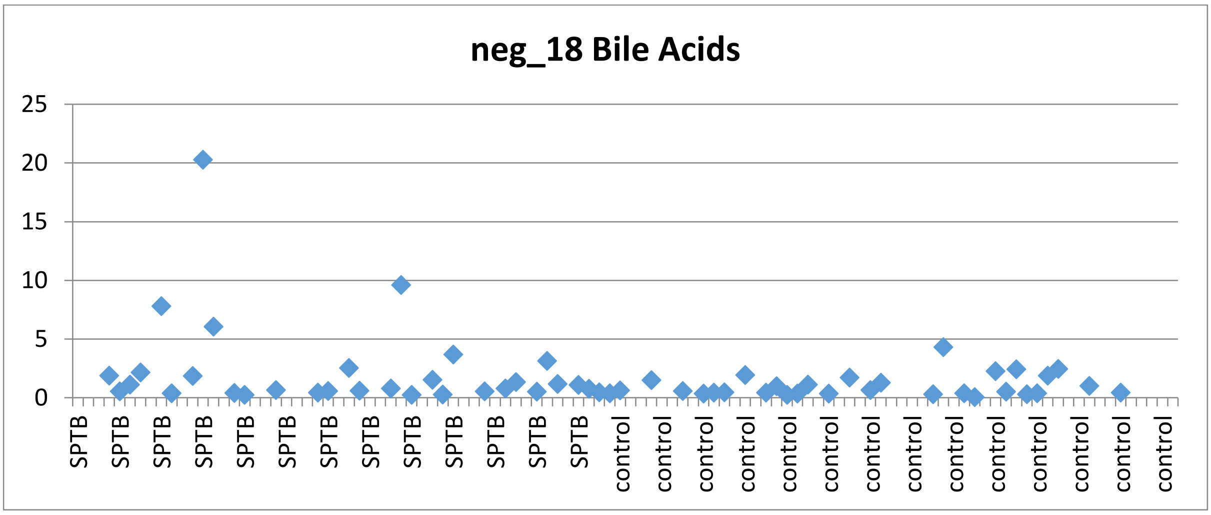

Figure S5: neg_19 at 15 weeks in cases and controls

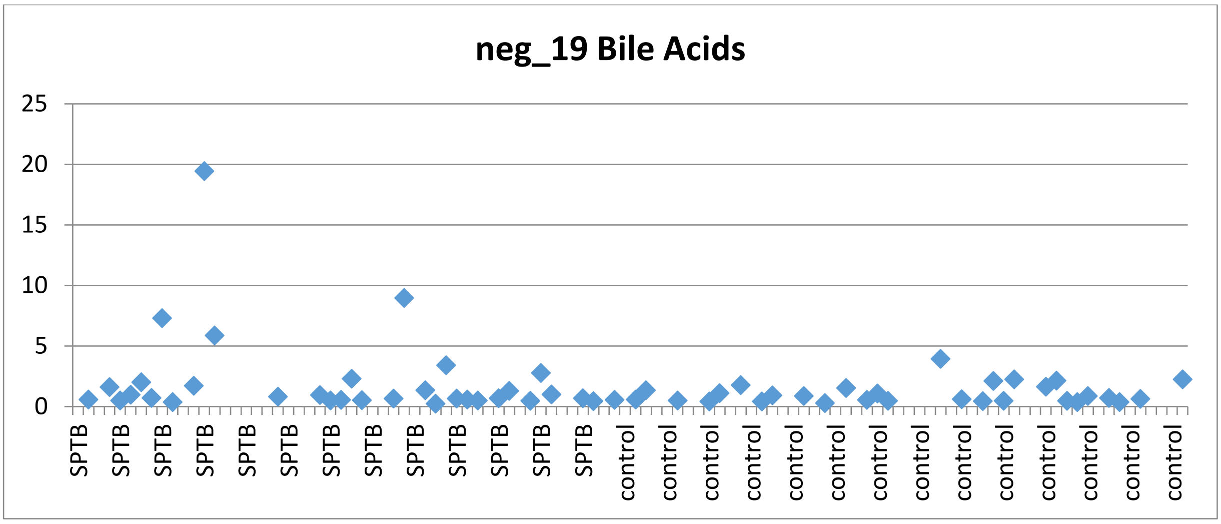

Figure S6: neg_20 at 15 weeks in cases and controls

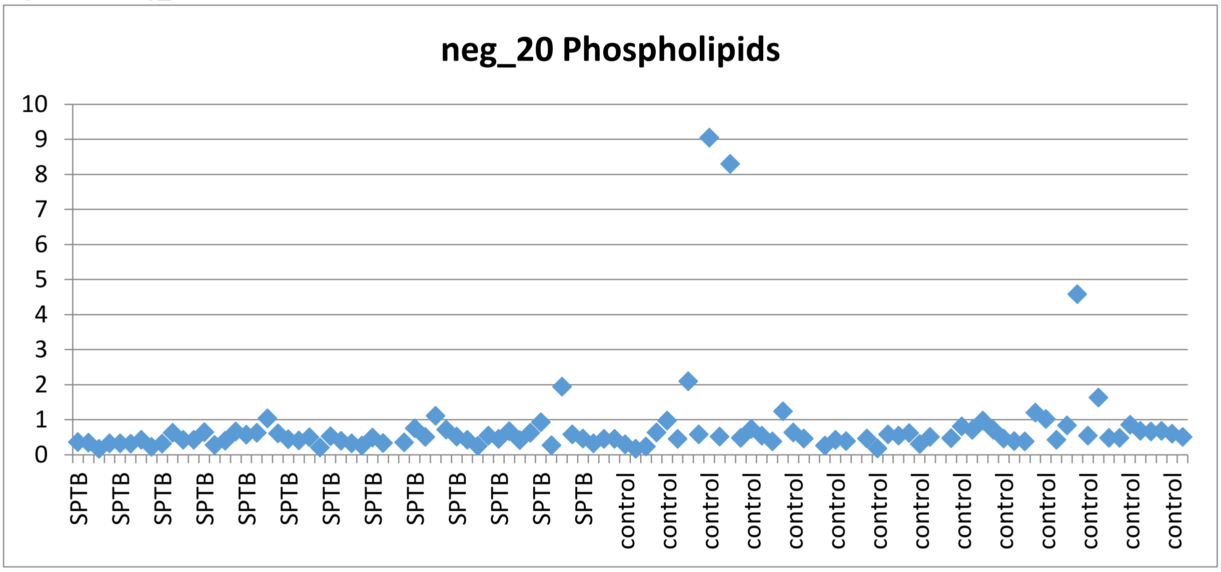

Figure S7: neg_21 at 15 weeks in cases and controls

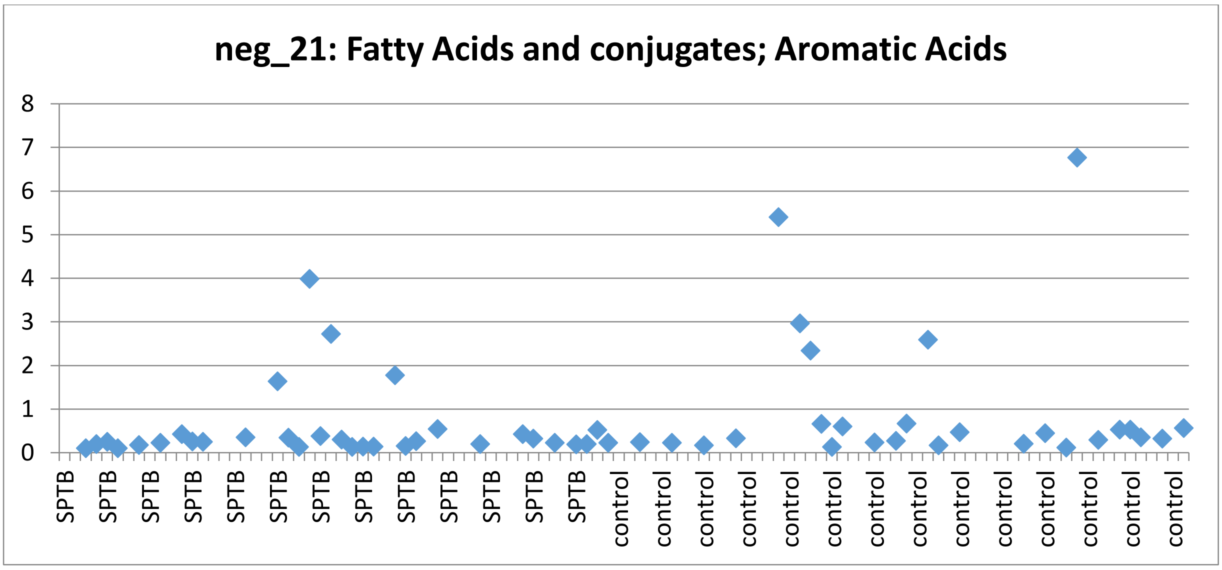

Figure S8: neg_22 at 15 weeks in cases and controls

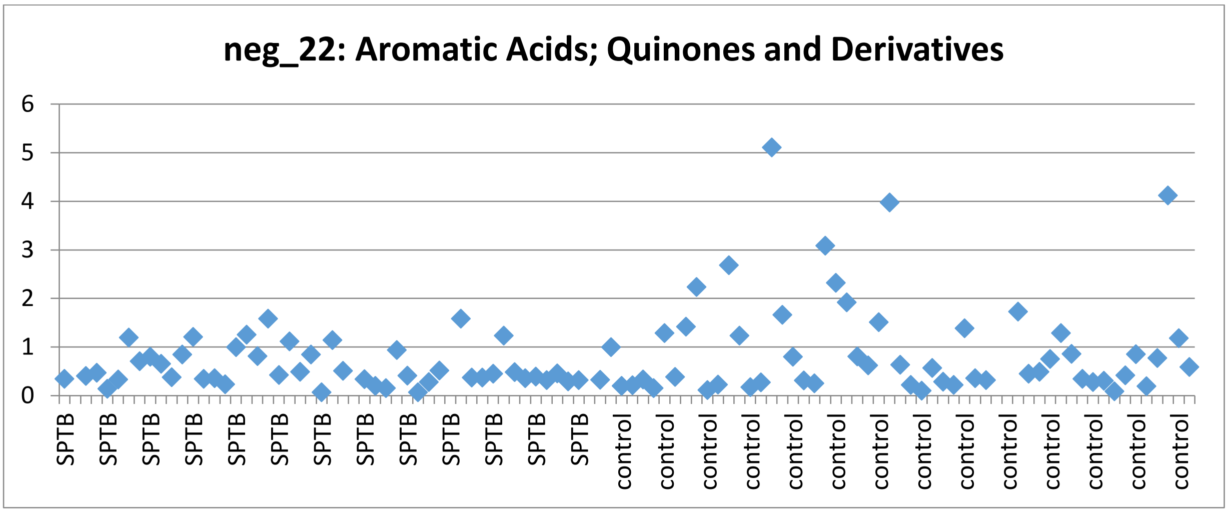

Figure S9: neg_23 at 15 weeks in cases and controls

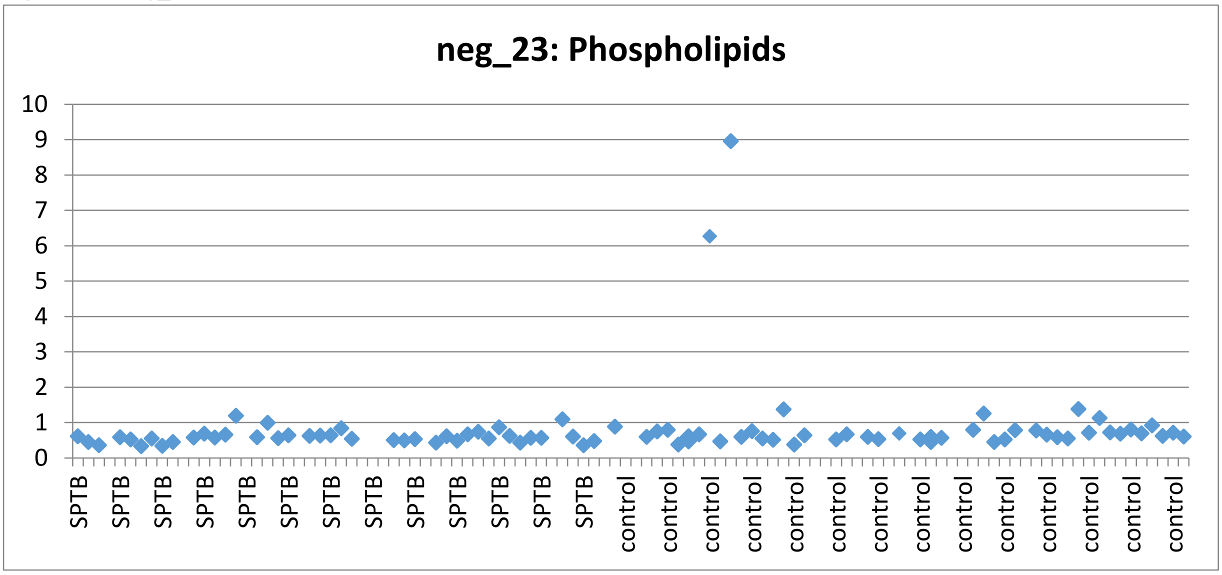

Figure S10: neg_24 at 15 weeks in cases and controls

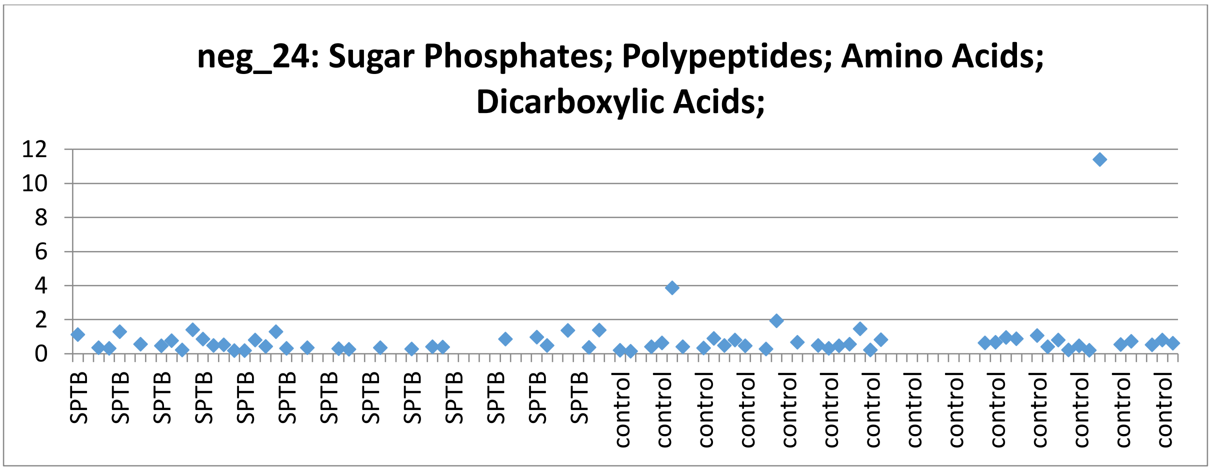

Figure S11: neg_25 at 15 weeks in cases and controls

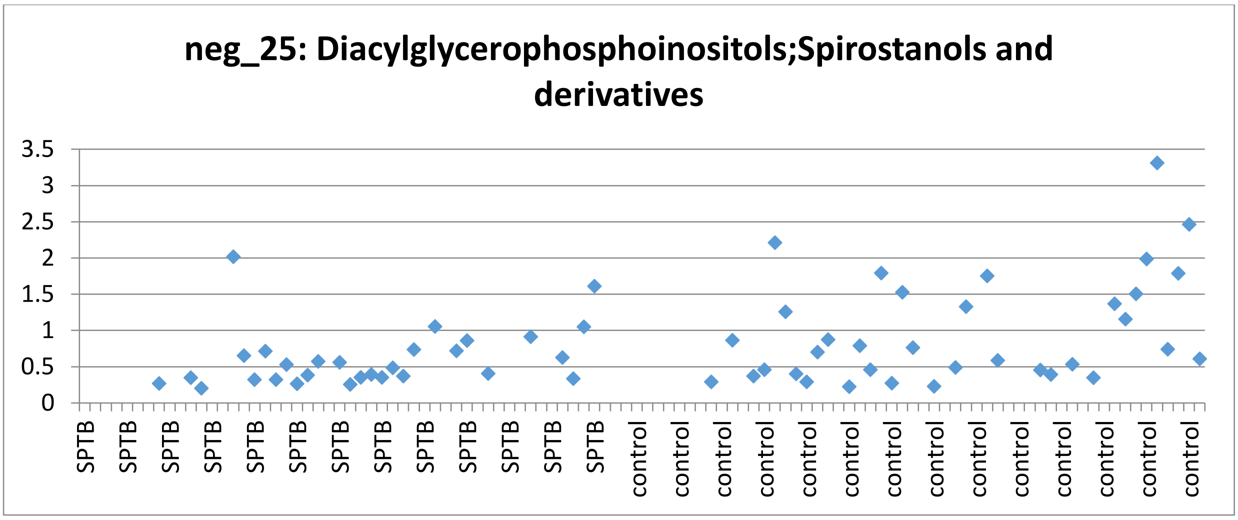

Figure S12: neg_26 at 15 weeks in cases and controls

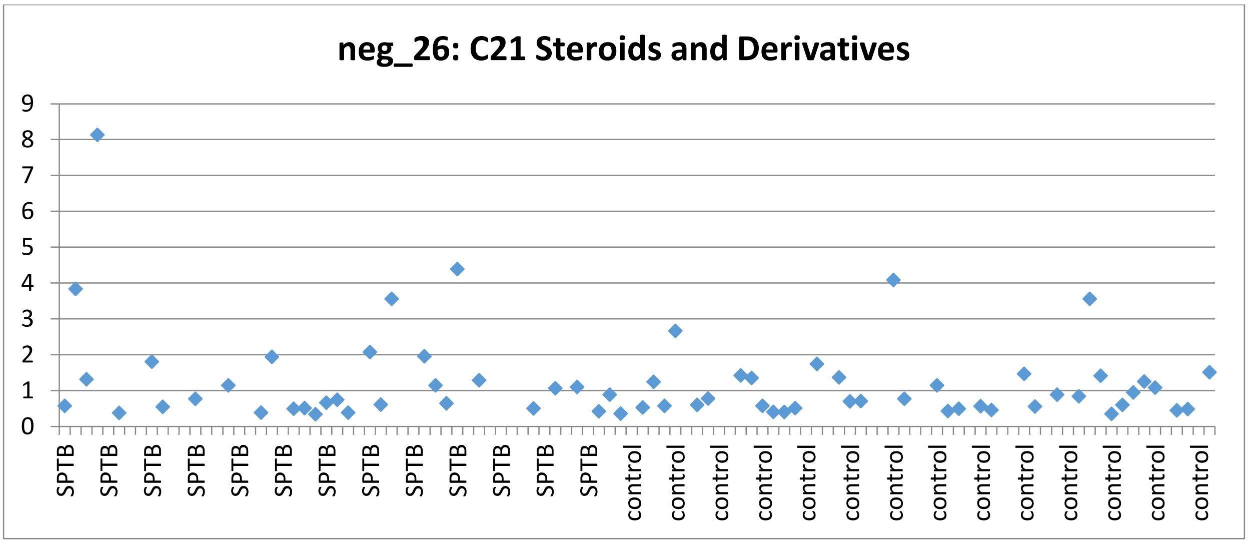

Figure S13: neg_27 at 15 weeks in cases and controls

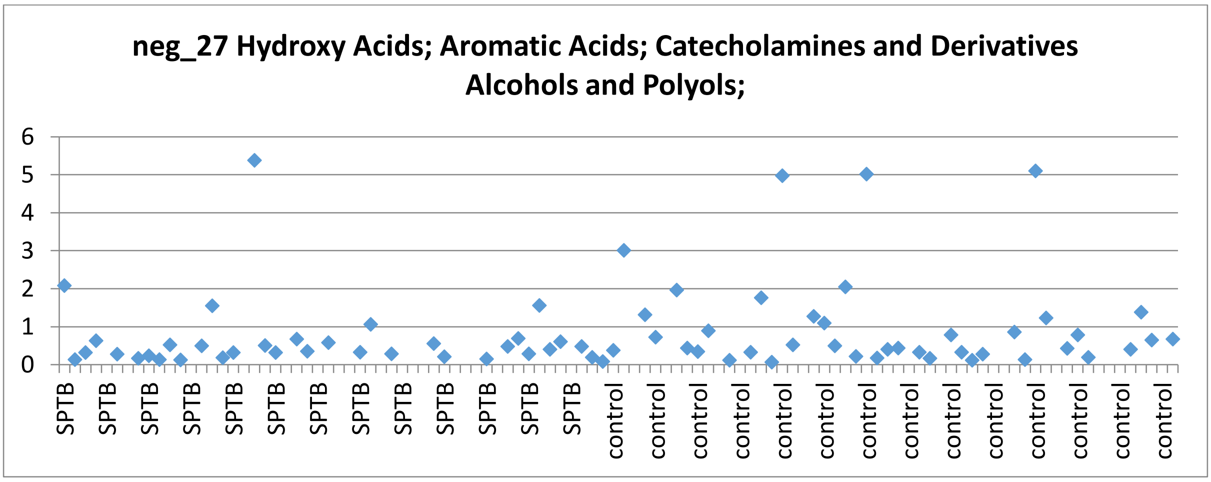

Figure S14: neg_28 at 15 weeks in cases and controls

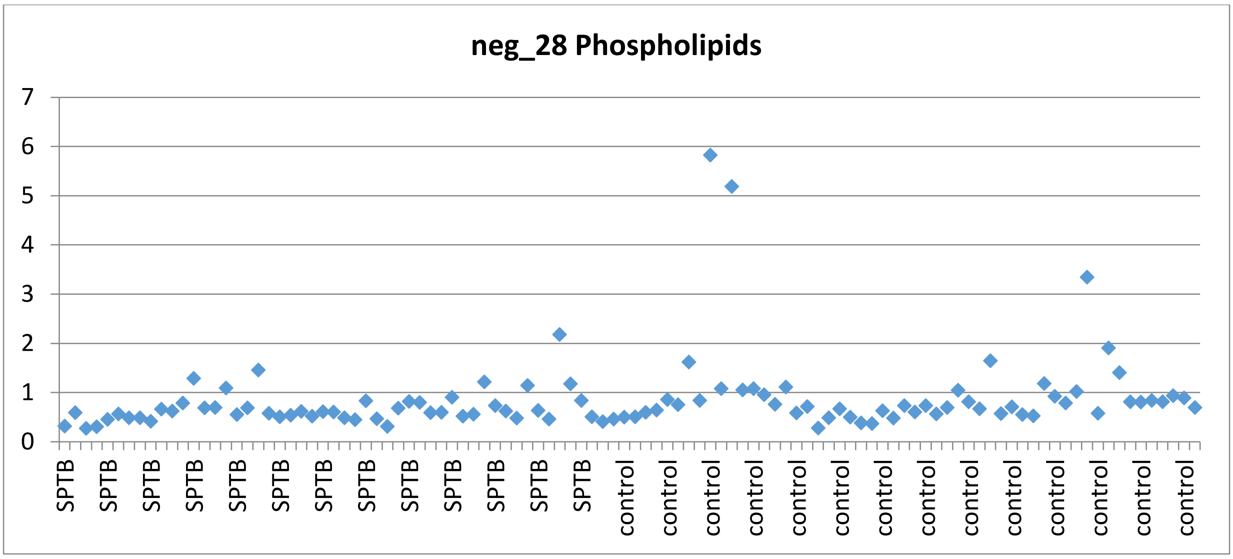

Figure S15: neg_29 at 15 weeks in cases and controls

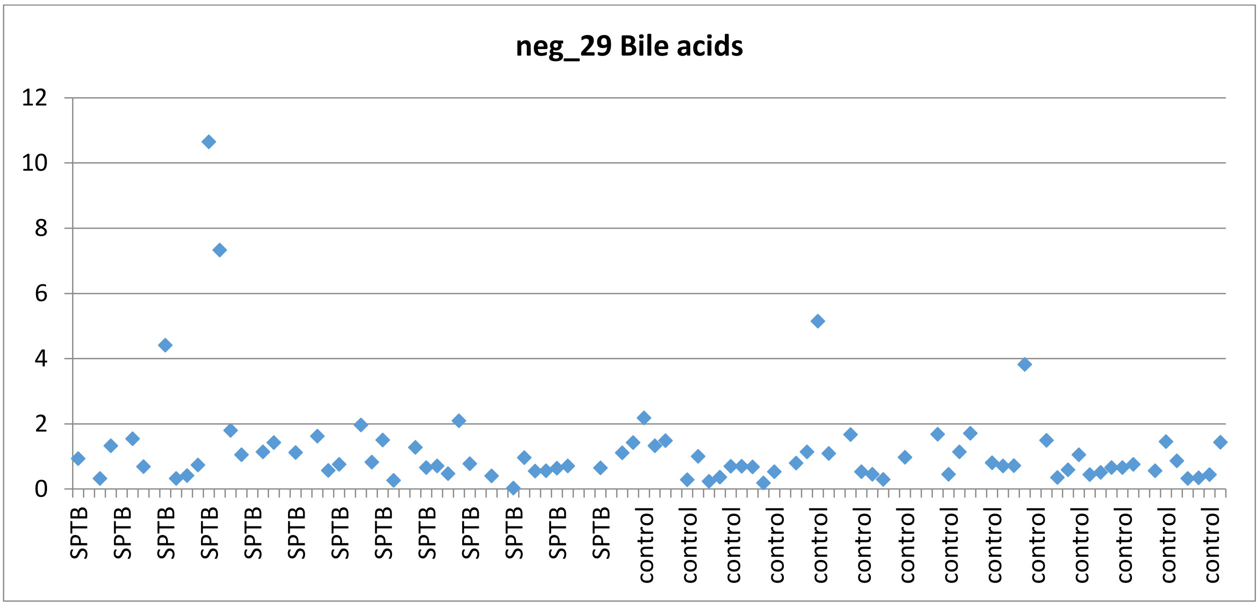

Figure S16: neg_30 at 15 weeks in cases and controls

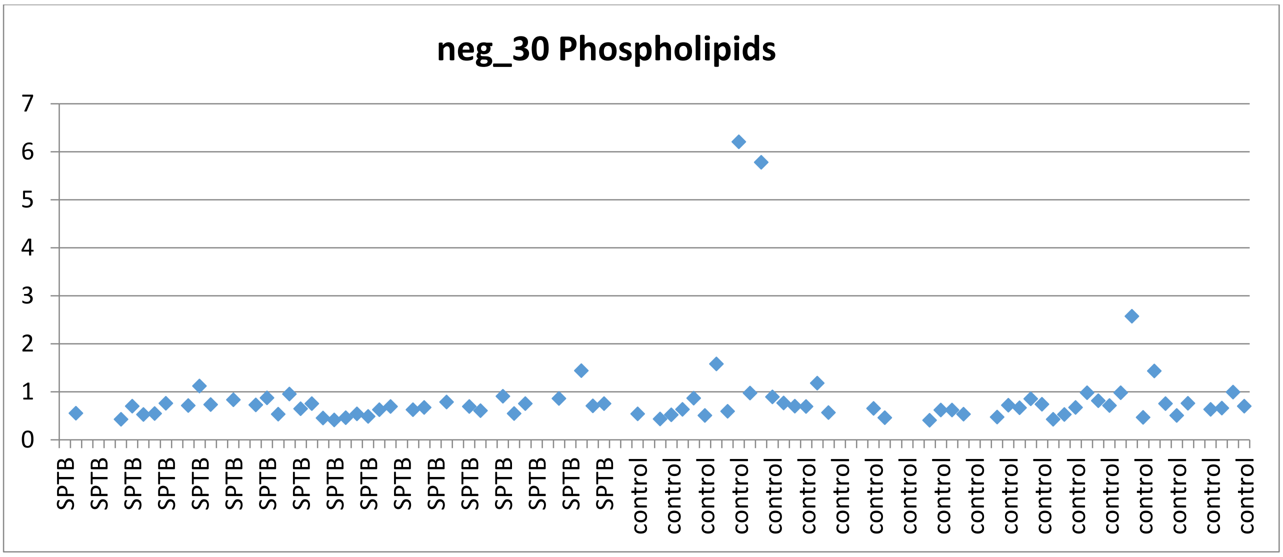

Figure S17: neg_31 at 15 weeks in cases and controls

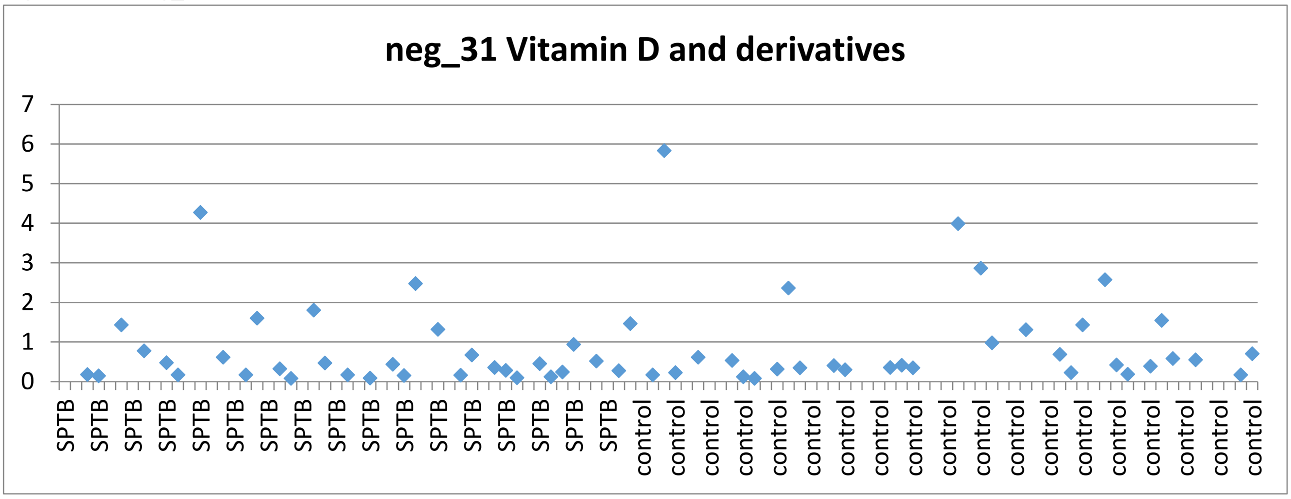

Figure S18: neg_32 at 15 weeks in cases and controls

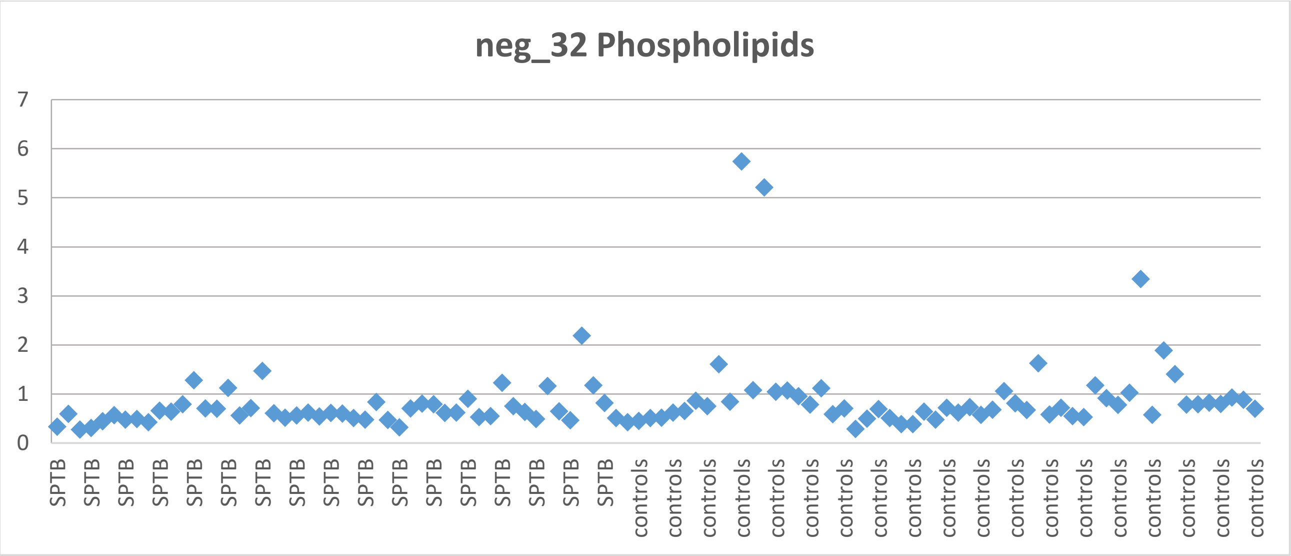

Figure S19: neg_33 at 15 weeks in cases and controls

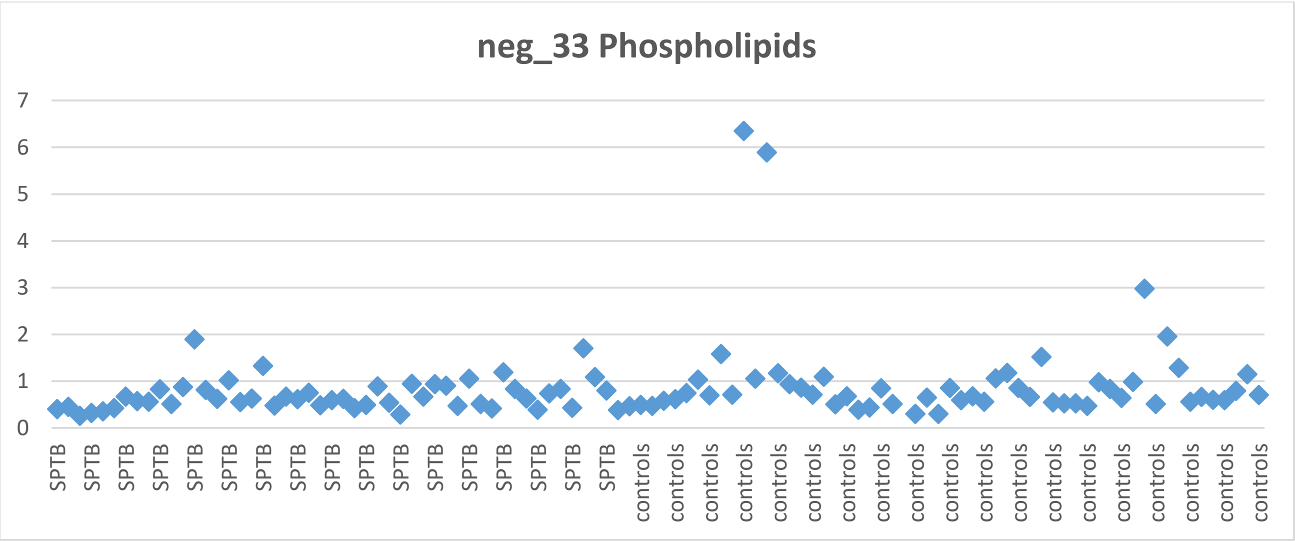

Figure S20: neg_34 at 15 weeks in cases and controls

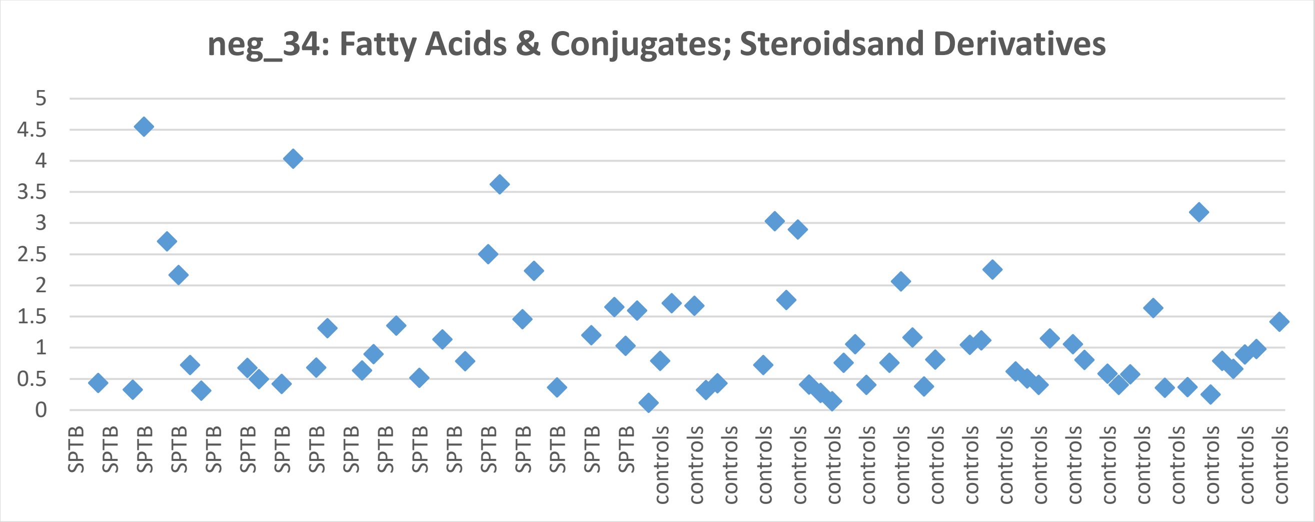

Figure S21: neg_35 at 15 weeks in cases and controls

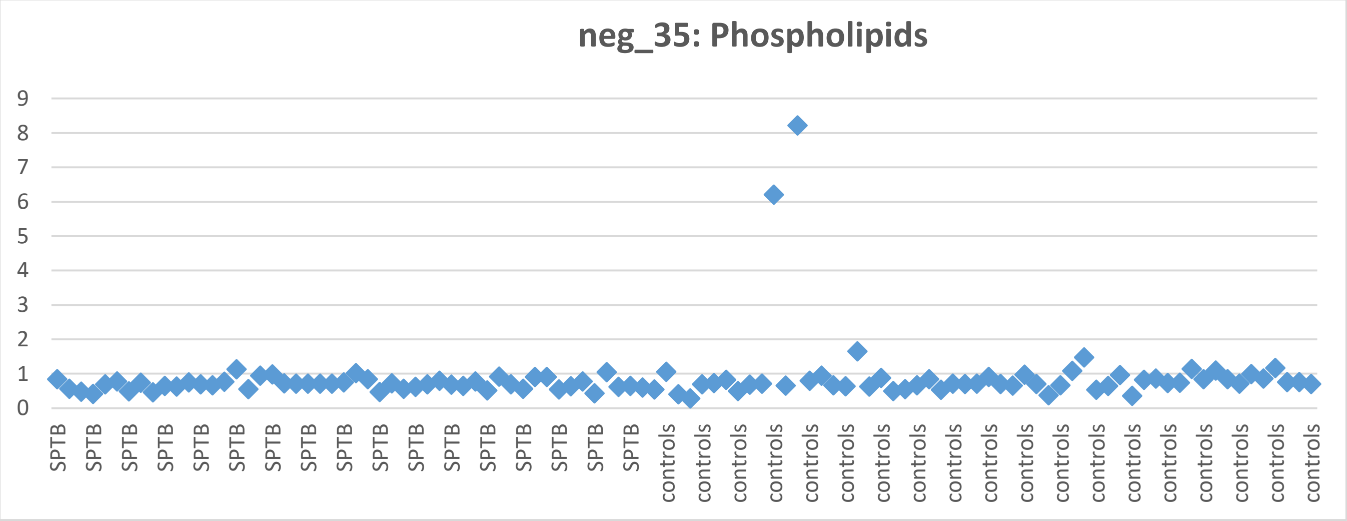

Figure S22: neg_36 at 15 weeks in cases and controls

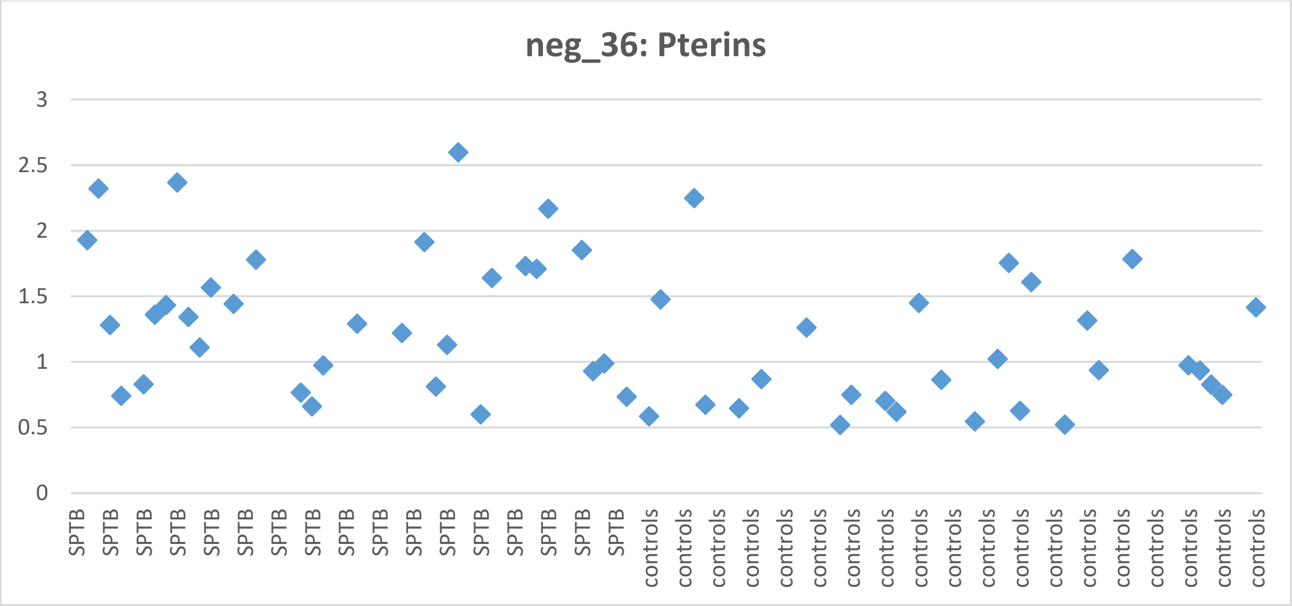

Figure S23: neg_37 at 15 weeks in cases and controls

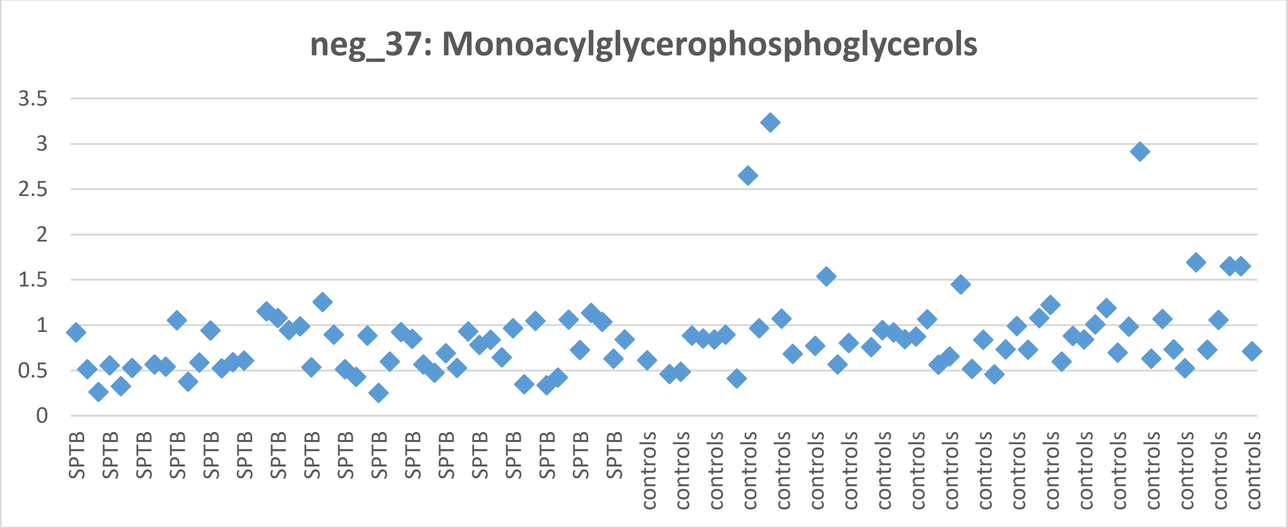

20 weeks gestational age dataset: Top features according to both Fold Change Ranking and PLSDA and VIP ranking preceded by separate imputation of cases and controls

Figure S24: neg_1 at 20 weeks in cases and controls

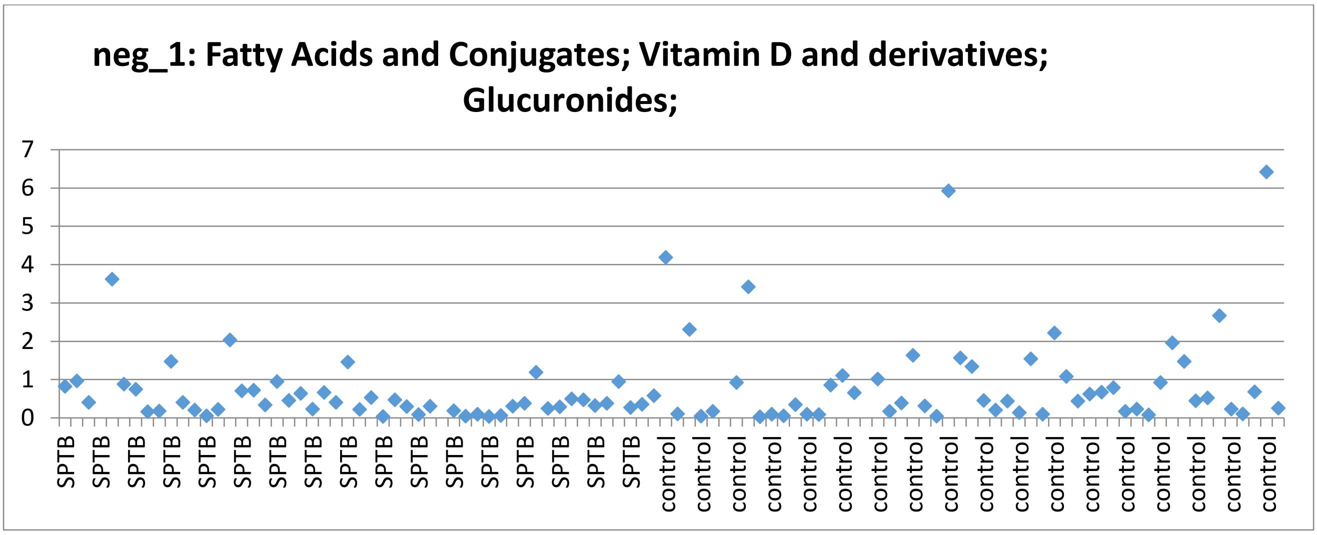

Figure S25: neg_2 at 20 weeks in cases and controls

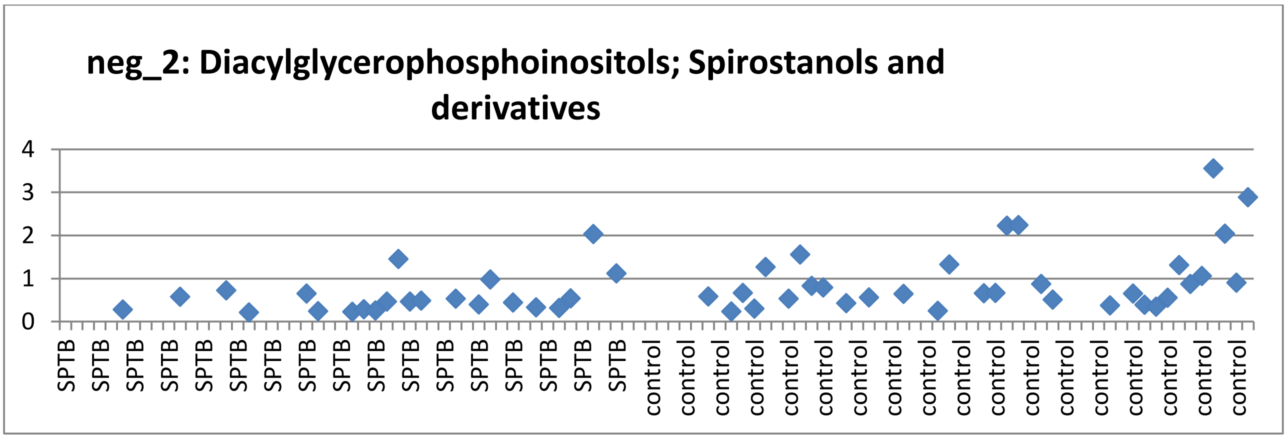

Figure S26: neg_3 at 20 weeks in cases and controls

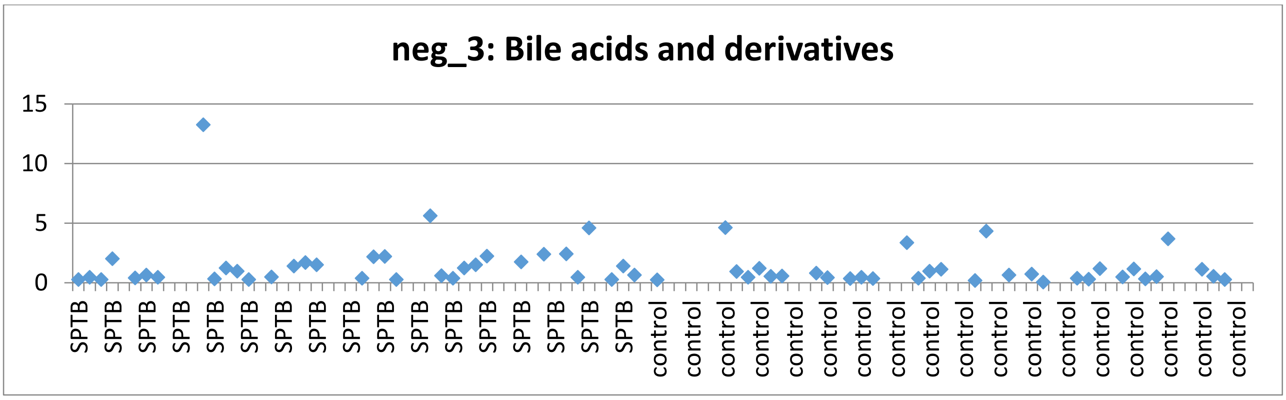

Figure S27: neg_4 at 20 weeks in cases and controls

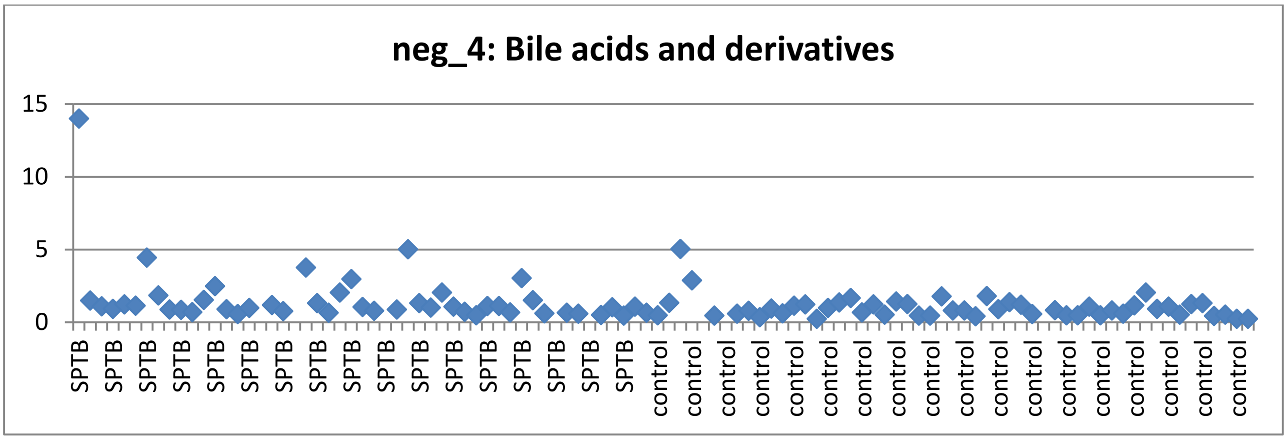

Figure S28: neg_5 at 20 weeks in cases and controls

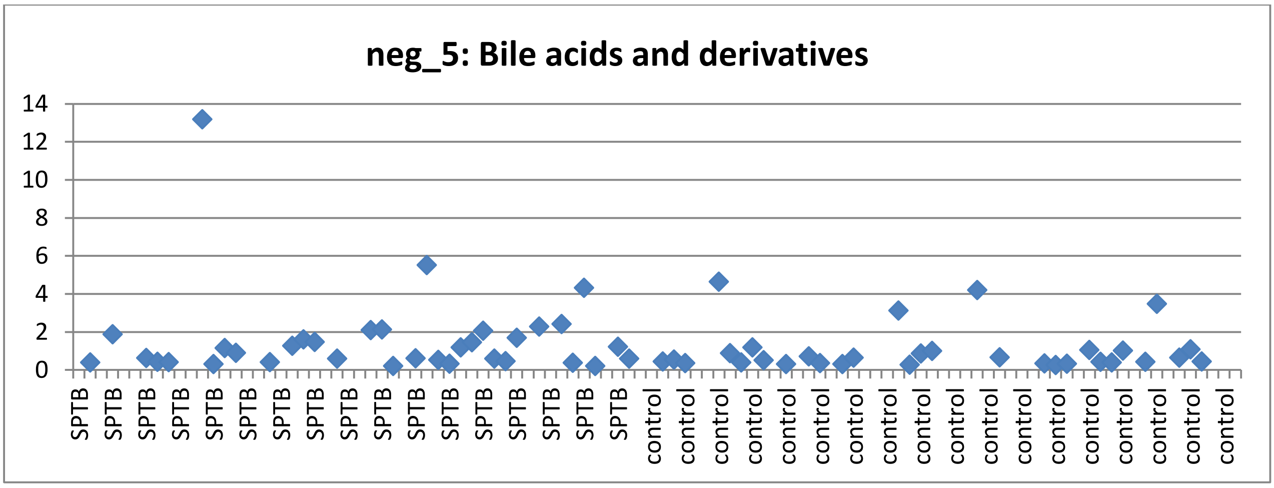

Figure S29: neg_6 at 20 weeks in cases and controls

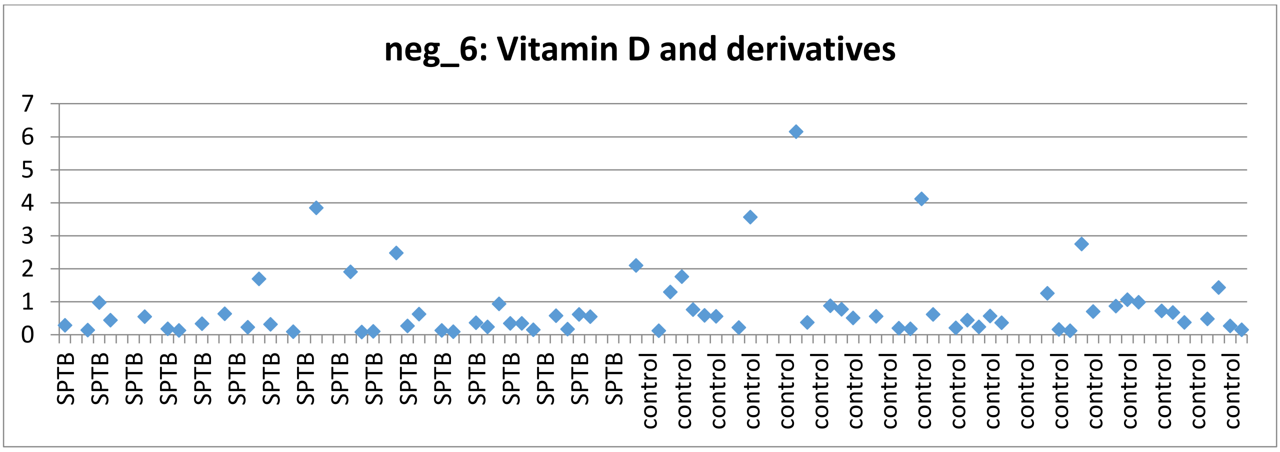

Figure S30: neg_7 at 20 weeks in cases and controls

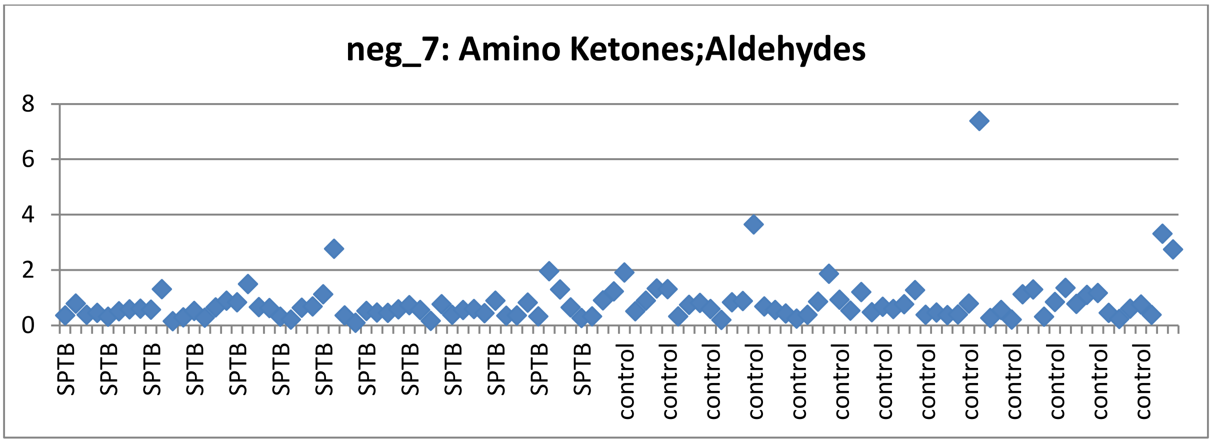

Figure S31: neg_8 at 20 weeks in cases and controls

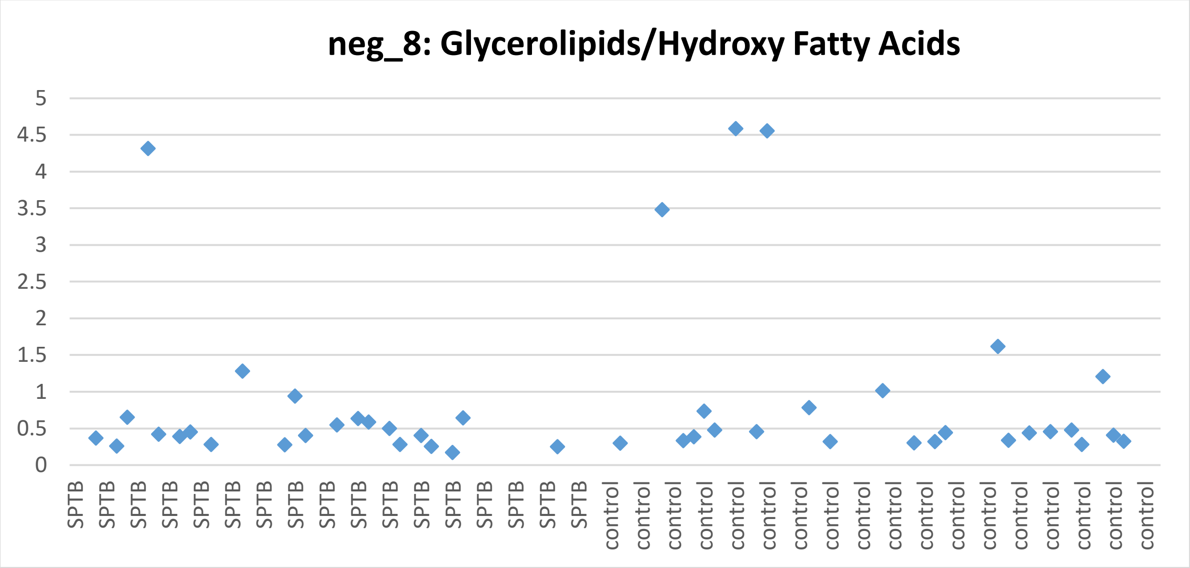

Figure S32: neg_9 at 20 weeks in cases and controls

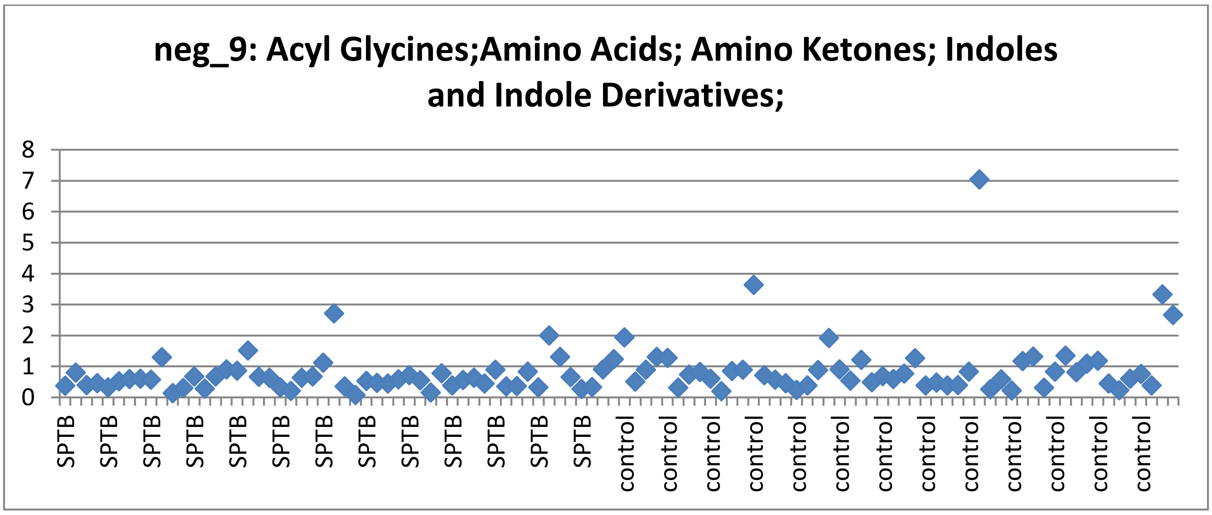

Figure S33: neg_10 at 20 weeks in cases and controls

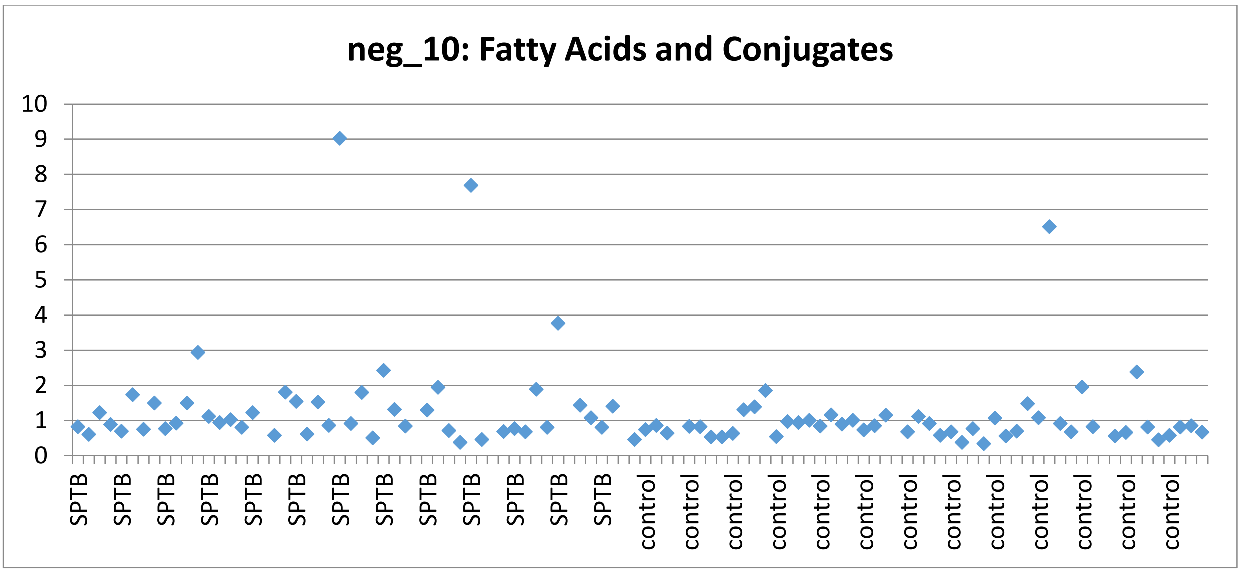

Figure S34: neg_11 at 20 weeks in cases and controls

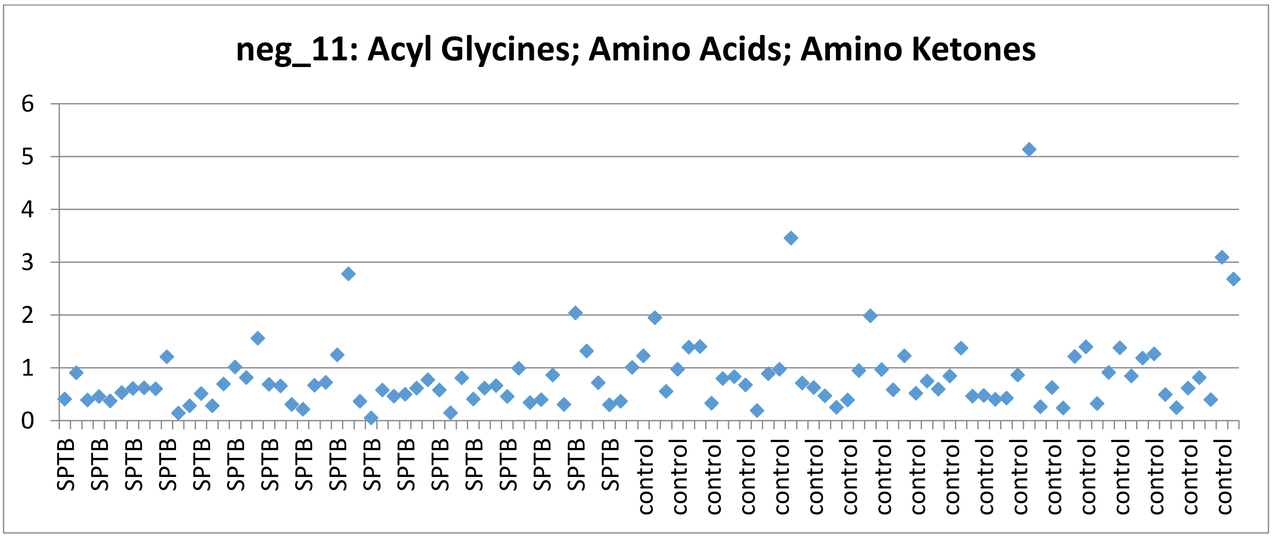

Figure S35: neg_12 at 20 weeks in cases and controls

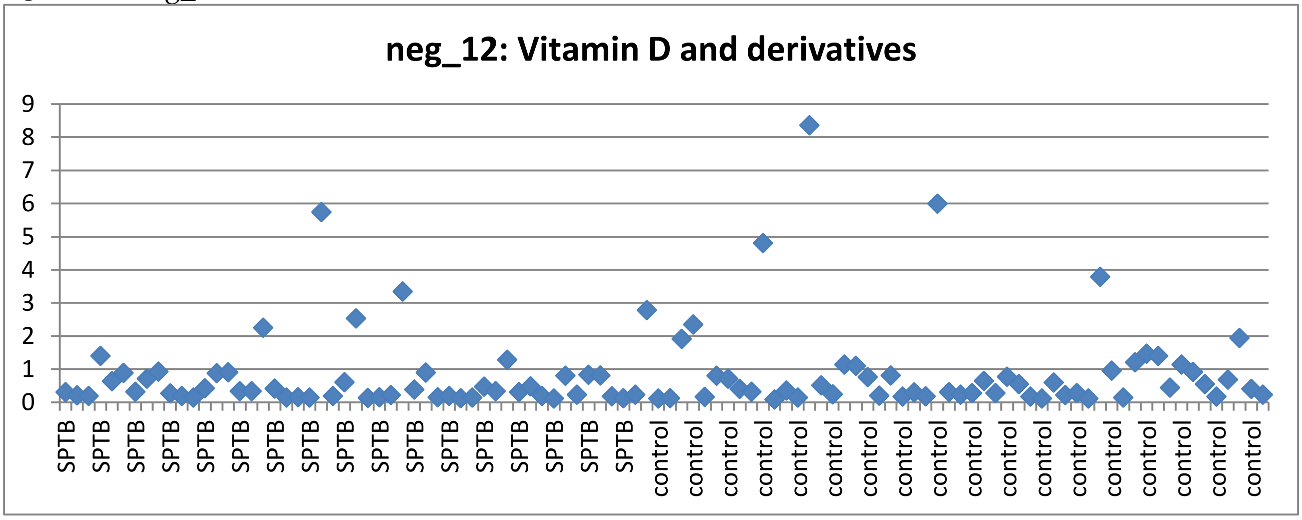

Figure S36: neg_13 at 20 weeks in cases and controls

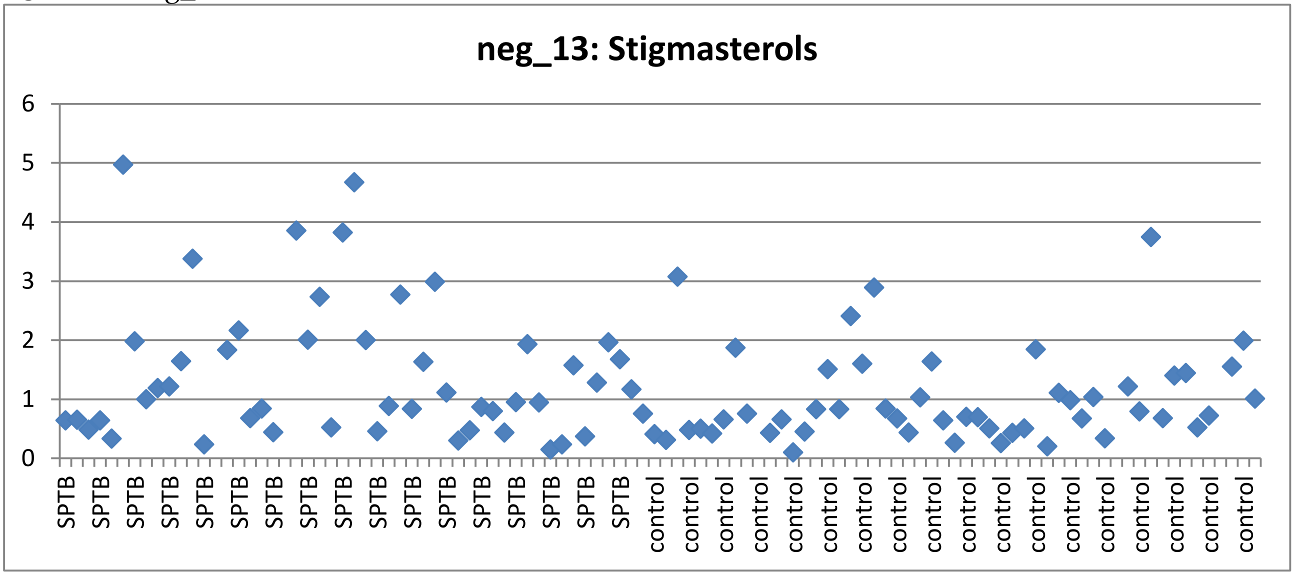

Figure S37: neg_14 at 20 weeks in cases and controls

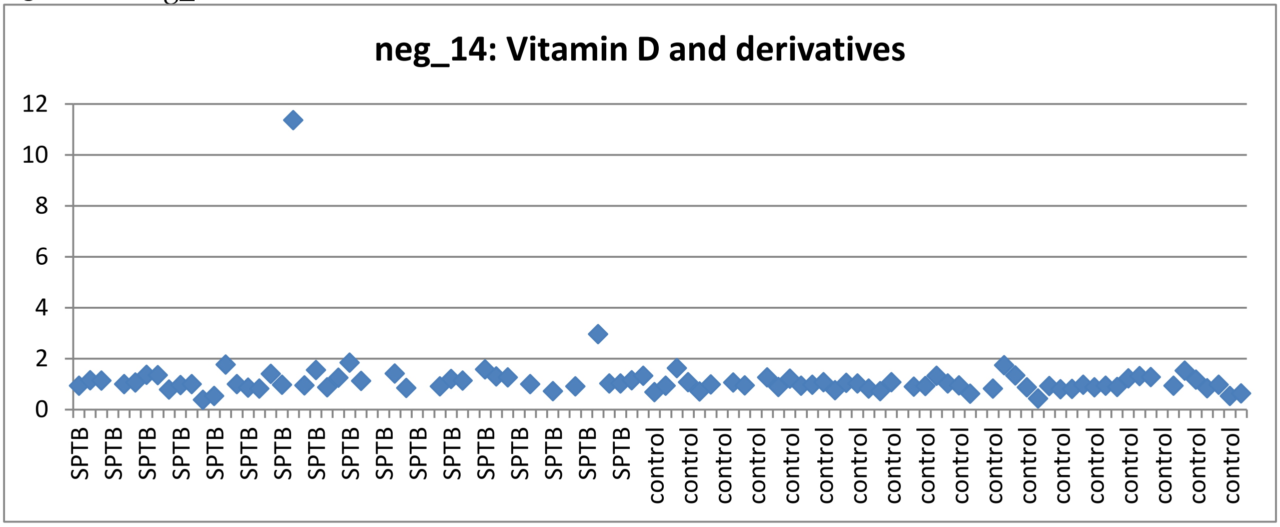

Figure S38: neg_15 at 20 weeks in cases and controls

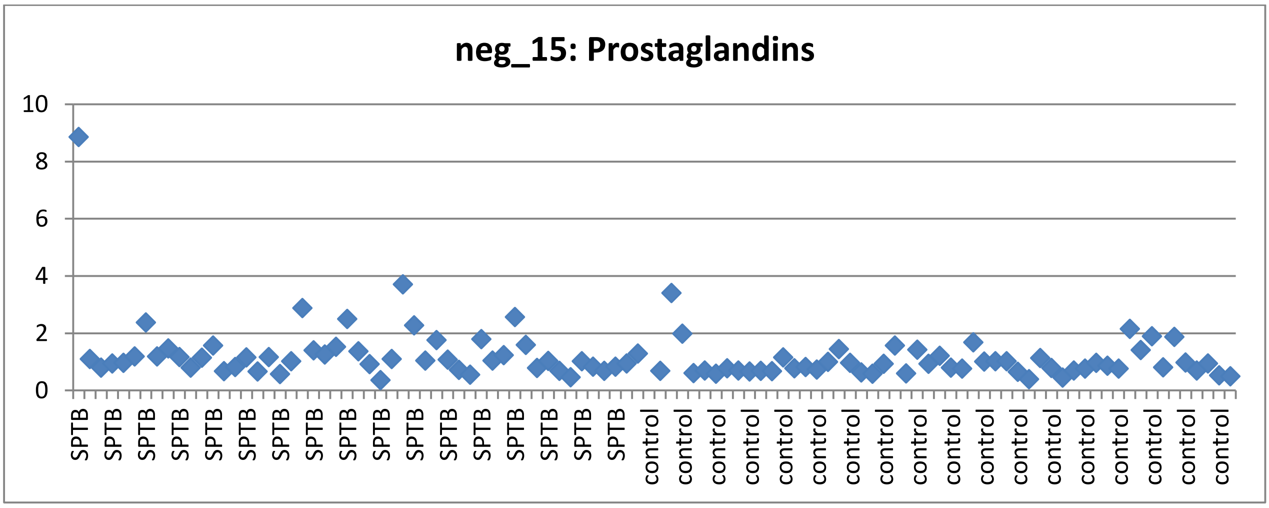

Figure S39: neg_16 at 20 weeks in cases and controls

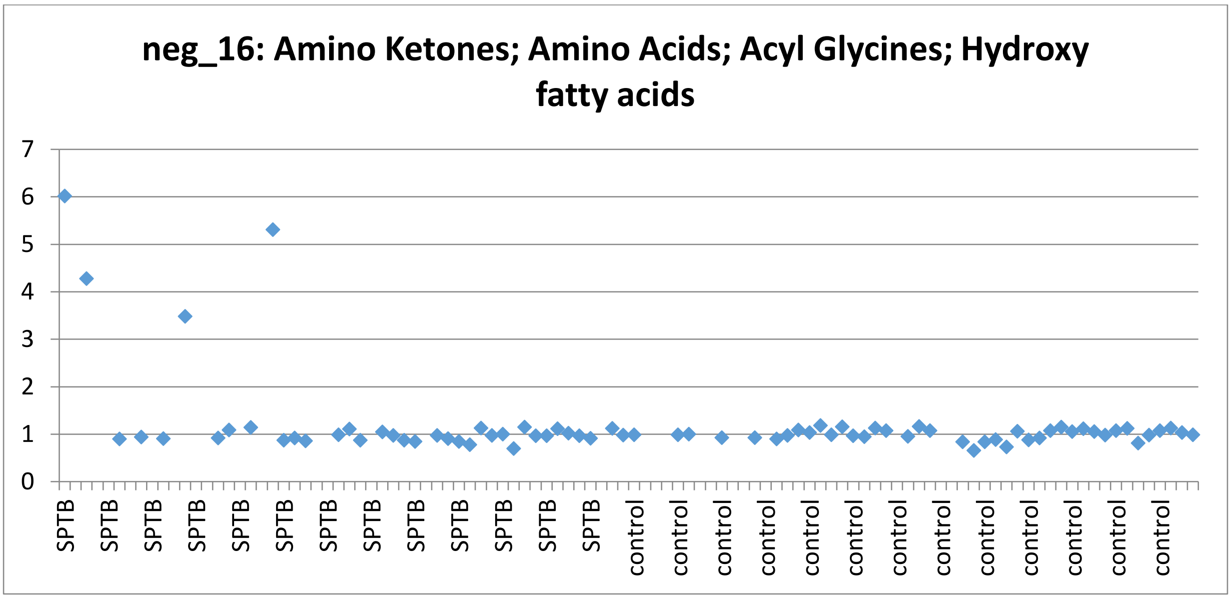

Figure S40: neg_17 at 20 weeks in cases and controls

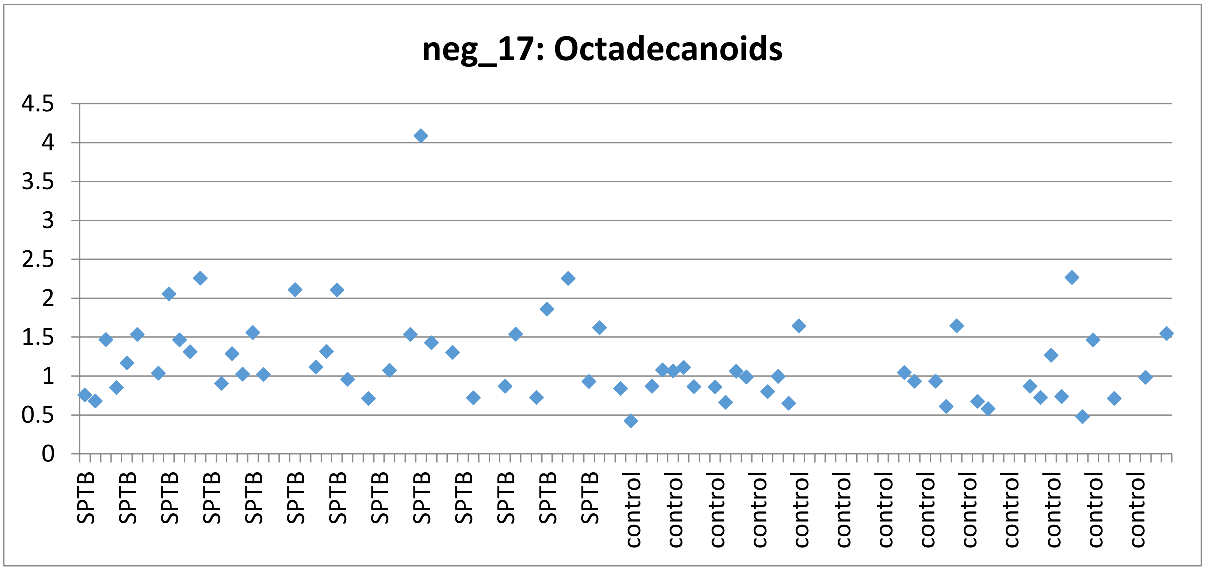

Table S4: Missing value % in 15 week dataset candidate lists and panels assessed by Panelomix

15 week dataset	Top 20 rank by FC	Top 20 rank MWU	Panel D	Panel E
CASES	24	9	3	2
CONTROLS	23	11	4	4
OVERALL	24	10	4	3

Table S5: Missing value % in 20 week dataset candidate lists and panels assessed by Panelomix

20 week dataset	Top 17 rank FC and PLSDA	Top 20 rank MWU	Panel A FC/PLSDA	Panel B FC/PLSDA	Panel C MWU
CASES	19	11	6	12	0.3
CONTROLS	18	11	4	8	0.6
OVERALL	18	11	5	10	0.5

Abbreviations:

AA: Amino Acids;
AG: Acyl Glycines;
AK: Amino Ketones;
ALC: Alcohols;
ALD: Aldehydes;
ARA: Aromatic Acids;
BA: Bile Acid;
CAT & D: Catecholamines and Derivatives;
C21-STR: C-21 Steroids
DA: Dicarboxylic Acids;
DIAG: Diacylglycerophosphoinositols;
FA: Fatty Acid;
FALD: Fatty Aldehydes;
GLU: Gluconorides;
GLP: Glycerolipids;
HA: Hydroxy Acids;
HFA: Hydroxy Fatty Acids;
IND: Indoles;
ISO: Isoprenoids
MOAG: Monoacylglycerophosphoglycerols
NA: Nucleic Acids;
N_ACLA: N_Acyl Amines;
N_GLSP: Neutral glycosphingolipids;
NUC: Nucleotides;
OCT: Octadecanoids;
PA: Polyamines;
PG: Prostaglandin;
PL: Phospholipds;
PO: Polyols
PP: Polypeptides;
PTR: Pterins;
PUR: Purines;
SP: Sugar Phosphates;
STG: Stigmasterols;

STR: Steroids;
SPM: Sphingomyelins;
VITD: Vitamin D and derivatives;

Table S6. Logistic Regression performed in Panelomix for the panels of biomarkers

**Panel**	**Dataset**<br><br>**(GA in weeks)**	**Selection method**	**% missing values**	**# features in final panel**	**pAUC**	**% Specificity**<br><br>**(95%CI)**	
Panel A	20	FC	4	5	9.2<br><br>(4.0-15.1)	70.9<br><br>(52.7-78.2)	79.6<br><br>(67.3-89.8)
Panel B	20	MWU	0.5	5	10.9 (6.9–14.9)	54.5<br><br>(41.8–67.3)	85.7<br><br>(75.5–93.9)
Panel C	15	FC and VIP	4	4	8.1<br><br>(4.9–11.9)	41.1<br><br>(28.6–53.6)	85.7<br><br>(75.5–93.9)
Panel D	15	MWU	3	4	8.9<br><br>(5.1–13.7)	62.5<br><br>(50.0–75.0)	75.5<br><br>(63.3–87.8)
